# Immune‐Cell Dynamics in the Tumor Microenvironment: Implications for Cancer Therapy

**DOI:** 10.1002/mco2.71040

**Published:** 2026-10-04

**Authors:** Pin Guo, Yizhao Xie, Rui Song, Mengdi Yang, Zhen Lin, Chao Guan, Zechen Wang, Jin Wen, Guang‐Liang Chen

**Affiliations:** ^1^ Department of Neurosurgery The Affiliated Hospital of Qingdao University Qingdao China; ^2^ Department of Medical Oncology, Department of Oncology Fudan University Shanghai Cancer Center, Shanghai Medical College, Fudan University Shanghai China; ^3^ Department of Rheumatology Shanghai Jiao Tong University School of Medicine Affiliated Renji Hospital and School of Biomedical Engineering Shanghai China; ^4^ Department of Medical Oncology, Cancer Center Zhejiang Provincial People's Hospital (Affiliated People's Hospital), Hangzhou Medical College Hangzhou China; ^5^ Department of Cardiovascular Diseases Mayo Clinic Rochester New York USA

**Keywords:** activator protein‐1, cancer immunotherapy, Fos‐like antigen 2, immune‐cell dynamics, transcriptional regulation, tumor microenvironment

## Abstract

Immune‐cell dynamics within the tumor microenvironment (TME) determine whether immune cells can reach, recognize, and sustain antitumor function against malignant cells under suppressive pressure. These dynamics are shaped by hypoxia, nutrient stress, cytokine and chemokine signaling, stromal architecture, vascular access, and multicellular crosstalk, and are further regulated by transcriptional and epigenetic programs. In this review, we organize TME immunity around four major barriers to effective therapy: failed immune access, defective antigen visibility and priming, failure to sustain effector function, and suppressive tissue remodeling. We use FOSL2/Fra‐2, a component of activator protein‐1 (AP‐1) transcription factor complexes, as an example of how local signals can produce distinct regulatory effects in malignant, immune, and stromal compartments. FOSL2‐associated programs have been linked to differentiation, plasticity, myeloid and stromal remodeling, stress adaptation, and selected immune‐regulatory processes; however, the strength and direction of these associations vary by lineage, tumor type, model system, and level of functional evidence. We distinguish perturbation‐supported mechanisms from correlative state signatures and identify the need for spatial validation, functional perturbation, and biomarker validation. This perspective supports biomarker‐guided TME‐directed therapy matched to the dominant immune barrier.

## Graphical abstract

Immune‐cell dynamics in the tumor microenvironment converge on four major barriers to effective therapy: failed immune access, defective antigen visibility and priming, loss of effector maintenance, and suppressive tissue remodeling. These barriers emerge from local hypoxic, cytokine, metabolic, and stromal signals. FOSL2/Fra‐2 provides a context‐dependent AP‐1 example of how shared signals can produce different regulatory effects across tumor, immune, and stromal compartments. Defining these compartment‐specific mechanisms may improve biomarker development and TME‐directed therapeutic strategies.

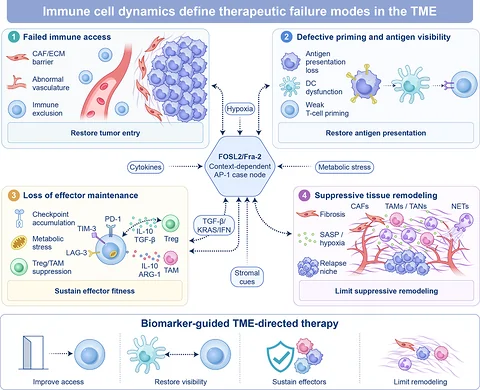

## 1 Introduction

Antitumor immunity fails not only because immune cells are absent, but also because they fail to reach, recognize, or eliminate malignant cells in the appropriate tissue context. The tumor microenvironment (TME) sets these conditions [1]. Malignant, stromal, vascular, and immune cells generate cytokine, hypoxic, metabolic, matrix‐derived, and contact‐dependent cues that reshape immune‐cell number, location, and function [1, 2, 3]. We refer to these coupled changes as immune‐cell dynamics, including recruitment, retention or exclusion, polarization, exhaustion, and acquisition of immunosuppressive or tissue‐remodeling states [1, 4, 5].

These dynamics help explain why immune‐cell abundance alone often fails to predict treatment response. An inflamed tumor may remain resistant if lymphocytes are trapped in the stromal regions, antigen presentation is ineffective, or effector cells lose fitness after tumor entry. Similar constraints shape responses to chemotherapy, targeted therapy, immune checkpoint blockade (ICB), oncolytic virotherapy, and cancer vaccination, each of which can alter the immune state and tumor‐cell killing [6, 7, 8, 9]. Single‐cell sequencing, spatial omics, and computational analysis have made these failure modes increasingly visible in human tumors [6, 7, 8]. However, visibility is not a mechanism. A central challenge is to define how signals within the TME are converted into durable immune states that support or restrict antitumor immunity [1, 2, 6, 7, 8, 10].

Transcriptional regulators provide a mechanistic link between extracellular signals and sustained changes in immune‐cell function [11]. The activator protein‐1 (AP‐1) transcription factor complex, formed by combinatorial dimers of FOS, JUN, ATF, and selected MAF family proteins, integrates stress, cytokine, and metabolic signaling across tumor and immune compartments [12, 13, 14, 15, 16]. Within this family, Fos‐like antigen 2 (FOSL2, also known as Fra‐2) is informative because it illustrates a broader problem in TME biology: the same transcriptional component can translate local stress signals into distinct biological programs in malignant, immune, and stromal cells [2, 10]. In contrast to c‐Fos and FosB, FOSL2 lacks their potent C‐terminal transactivation domain, making its transcriptional activity particularly dependent on dimer composition and the surrounding regulatory context [13, 17, 18, 19, 20]. Partner availability, chromatin accessibility, and upstream signaling can therefore alter FOSL2 genomic occupancy and transcriptional activity, allowing the same factor to regulate different gene programs across cell states.

FOSL2 is broadly expressed across mammalian tissues and is required for normal development and postnatal viability [12, 21, 22]. In cancer, its activity extends beyond malignant cells to immune and stromal compartments. In malignant cells, FOSL2 has been linked to proliferation, invasion, and metastasis [16, 20, 23], whereas in immune and stromal cells, it has been associated with lineage plasticity, immune recognition, extracellular matrix (ECM) remodeling, and niche organization [20, 24, 25, 26, 27, 28, 29, 30, 31, 32]. These context‐dependent effects argue against a uniform pro‐ or antitumor role for FOSL2. Its function likely depends on cell type, transcriptional partners, and tissue context.

In this review, we conceptualize immune‐cell dynamics as the spatial organization, functional states, and regulatory interactions that shapes antitumor immunity and therapeutic responses. We organize these dynamics into four common therapeutic failure modes: failed immune access, defective antigen visibility and priming, loss of effector maintenance, and suppressive tissue remodeling (Table 1). We propose that these failure modes provide a useful framework for interpreting TME biology and guiding combination therapies. Immune‐cell abundance or subset labels alone may be insufficient. The role of FOSL2/Fra‐2 is context dependent, and its contribution to tumor progression and therapeutic vulnerability varies across cellular and disease settings. We also distinguish perturbation‐supported mechanisms from correlative state signatures and highlight priorities for spatial validation, functional perturbation, and biomarker validation.

**TABLE 1:** Immune‐dynamic failure modes in the tumor microenvironment.

Failure mode	Tumor phenotype	Core mechanism	Therapeutic logic
Failed immune access	Effector cells remain outside tumor nests or accumulate at stromal margins.	Abnormal vessels, dense ECM, CAF barriers, chemokine misdirection, and hypoxia‐associated exclusion [33, 34, 35, 36, 37, 38, 39, 40].	Restoration of immune‐cell access to tumor nests.
Defective antigen visibility and priming	Immune cells are present, but antigen presentation and T‐cell priming are weak.	MHC‐I/APM loss, impaired IFN signaling, dysfunctional or suppressive DC states, and poor costimulation [41, 42, 43, 44, 45, 46, 47, 48, 49].	Restoration of antigen visibility, DC licensing, and productive T‐cell priming.
Failure to sustain effector function	Effector lymphocytes enter the tumor but become exhausted, metabolically impaired, or functionally suppressed.	Chronic antigen exposure, checkpoint accumulation, mitochondrial stress, lactate/acidosis, adenosine, and Treg/myeloid suppression [50, 51, 52, 53, 54, 55, 56, 57, 58].	Preservation or restoration of effector fitness after tumor entry.
Suppressive tissue remodeling	Therapy or tumor progression creates fibrotic, hypoxic, myeloid‐rich, or NET‐rich tumor niches that constrain antitumor immunity.	CAF activation, TAM/TAN remodeling programs, SASP, NETs, vascular dysfunction, and hypoxia‐redox stress [36, 59, 60, 61, 62, 63, 64, 65, 66, 67].	Prevention or reversal of suppressive tissue remodeling and maintenance of an immune‐permissive tissue architecture.

*Note*: These failure modes are not mutually exclusive and may coexist within the same tumors.

Abbreviations: APM, antigen‐processing machinery; CAF, cancer‐associated fibroblast; DC, dendritic cell; ECM, extracellular matrix; IFN, interferon; MHC‐I, major histocompatibility complex Class I; NET, neutrophil extracellular trap; SASP, senescence‐associated secretory phenotype; TAM, tumor‐associated macrophage; TAN, tumor‐associated neutrophil; TME, tumor microenvironment; Treg, regulatory T cell.

## 2 TME Compartments and Spatial‐Temporal Dynamics

The TME is organized into spatial niches that evolve during tumor progression and treatment. Immune, stromal, vascular, and metabolic features of the TME collectively influence tumor growth, immune escape, and therapeutic response (Figure 1) [68, 69, 70]. Tumors contain spatially distinct immune niches in which local tissue conditions shape immune‐cell behavior. Perivascular regions, invasive margins, tertiary lymphoid structures (TLSs), and poorly vascularized or hypoxic areas provide different levels of vascular access, extracellular matrix constraint, nutrients, metabolites, and cytokine signals. These spatially restricted cues regulate immune‐cell recruitment, retention, activation, and effector capacity, making cellular location an important determinant of antitumor immunity [70, 71, 72, 73, 74]. CD8^+^ T cells can be excluded from tumor nests by collagen‐rich, fibroblast‐dense stroma or accumulate in perivascular regions with dysfunctional features in selected settings [75]. Regulatory T cells (Tregs) and B cells localize within or around TLSs, whose immune effects depend on their cellular composition and maturation state [70, 71]. Myeloid cells, including tumor‐associated macrophages (TAMs), dendritic cells (DCs) and myeloid‐derived suppressor cell (MDSC)‐like populations, alter their transcriptional and metabolic states in response to hypoxia, extracellular acidity, and local cytokine gradients (Figure 1A,B) [50, 68, 70, 76].

**FIGURE 1:**
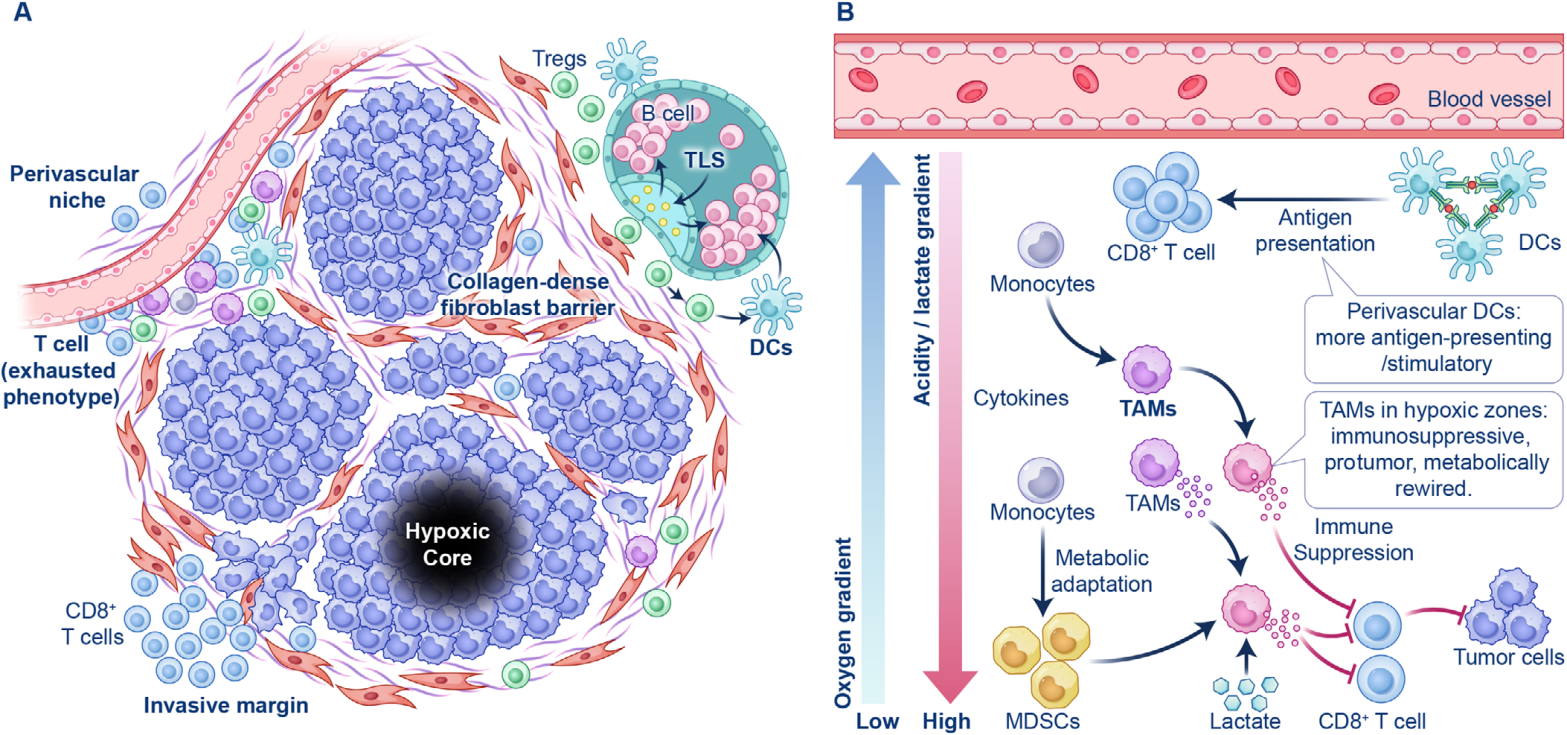
Spatial and metabolic organization of immune niches in the tumor microenvironment. (A) The tumor microenvironment (TME) contains distinct immune niches, including perivascular regions, invasive margins, tertiary lymphoid structures (TLSs), and hypoxic cores. CD8^+^ T cells may accumulate at the invasive margins, whereas collagen‐dense fibroblast barriers restrict their penetration into tumor nests. Regulatory T cells (Tregs), B cells, and dendritic cells (DCs) localize within or around TLSs, illustrating spatially organized immune compartmentalization. (B) Oxygen and lactate/acidity gradients shape immune cell states across the TME. Perivascular DCs can retain antigen‐presenting and stimulatory features, whereas tumor‐associated macrophages (TAMs) enriched in hypoxic regions can adopt metabolically rewired, immunosuppressive, and protumor states. These niche‐dependent myeloid programs, along with cytokine signaling and lactate accumulation, can suppress CD8^+^ T‐cell function and reinforce local immunosuppression. DC, dendritic cell; MDSC, myeloid‐derived suppressor cell; TAM, tumor‐associated macrophage; TLS, tertiary lymphoid structure; TME, tumor microenvironment; Treg, regulatory T cell.

Beyond these cell‐specific niches, ECM density, perfusion, interstitial pressure, and tissue mechanics shape how immune cells move through and gain access to tumors [76, 77, 78, 79]. This spatial organization is dynamic: tumor progression and therapy can shift tumors between inflamed and excluded states and reshape local immune architecture [80, 81]. Spatiotemporal analysis can therefore reveal where antitumor immunity breaks down, from vascular entry and stromal transit to priming and postentry effector function [82]. These spatial patterns are most informative when linked to functional evidence of impaired immune activity.

## 3 Local Interface, Cell‐State, Tumor‐Intrinsic, and Distal Regulators of Tumor Immune Set‐Points

This section summarizes the major local, cell‐state, tumor‐intrinsic, and systemic regulators of immune states in the TME. FOSL2/Fra‐2 is used as a cross‐compartment example of context‐dependent transcriptional regulation.

### 3.1 Local Interface Regulatory Axes of Tumor Immune Modulation

#### 3.1.1 Antigen Presentation and Interferon Signaling Axis

Productive antitumor immunity depends on the presentation of tumor‐derived peptides by major histocompatibility complex (MHC) Class I molecules, allowing CD8^+^ T cells to recognize malignant cells [41]. Under immune selection pressure, tumors can reduce antigen presentation through genetic or epigenetic disruption of the antigen processing and presentation machinery [42]. Interferon (IFN) signaling is a major regulator of this process. Type I IFNs, which can be induced by nucleic acid–sensing pathways including cGAS–STING, promote DC activation and cross‐priming and enhance MHC I antigen presentation. IFN‐γ likewise increases antigen processing and MHC I expression, but sustained signaling can also induce programmed death‐ligand 1 (PD‐L1) and other inhibitory programs that promote adaptive immune resistance [43]. Thus, the same inflammatory signals that improve tumor recognition can also engage feedback pathways that limit T‐cell activity (Figure 2A) [43, 44]. Microenvironmental stress can further disrupt antigen processing and presentation, providing an additional route to immune escape [43, 45].

**FIGURE 2:**
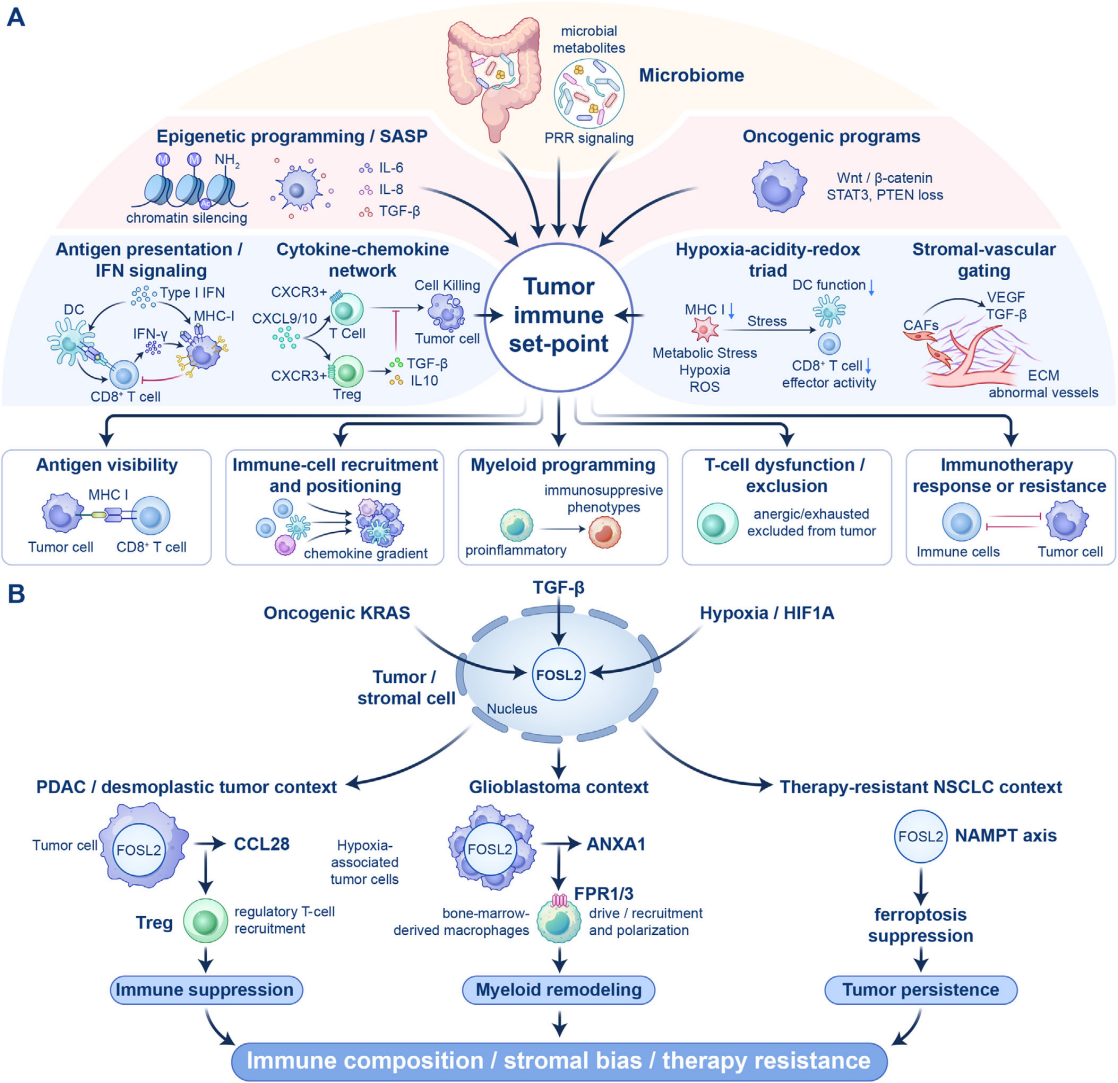
Multiscale regulators of tumor immune set‐points. (A) The tumor immune set‐point is shaped by convergent local, tumor‐intrinsic, and systemic inputs. Local interface signals, including antigen presentation, interferon signaling, cytokine–chemokine networks, hypoxia–acidity–redox stress, and stromal–vascular gating, regulate antigen visibility, immune‐cell recruitment and positioning, myeloid programming, and T‐cell dysfunction or exclusion. Cell‐state and tumor‐intrinsic programs, including epigenetic remodeling, senescence‐associated secretory phenotype (SASP) programs, and oncogenic pathways, further tune immune responsiveness. Distal microbial cues and metabolites can modify dendritic cell function, myeloid states, and effector–regulatory T‐cell balance, thereby influencing response or resistance to immunotherapy. (B) FOSL2 (FOS like 2, AP‐1 transcription factor subunit), which encodes Fra‐2, is a context‐dependent transcriptional node linking oncogenic and stress cues to distinct immune and stromal outputs. In desmoplastic tumors, such as pancreatic ductal adenocarcinoma (PDAC), KRAS‐FOSL2 signaling promotes CCL28‐associated regulatory T‐cell (Treg) recruitment. In glioblastoma, hypoxia‐associated HIF1A‐FOSL2 signaling induces ANXA1 expression and supports FPR1/3‐dependent recruitment and polarization of bone‐marrow‐derived macrophages. In therapy‐resistant non–small‐cell lung cancer (NSCLC), FOSL2‐NAMPT signaling is associated with ferroptosis resistance and tumor persistence. These examples position FOSL2 as a context‐specific biasing module for immune composition, stromal remodeling, and therapy resistance, rather than a universal master regulator of tumor immunity. ANXA1, annexin A1; CCL28, C‐C motif chemokine ligand 28; FOSL2, FOS‐like 2 AP‐1 transcription factor subunit; FPR1/3, formyl peptide receptor 1/3; HIF1A, hypoxia‐inducible factor 1 subunit alpha; NAMPT, nicotinamide phosphoribosyltransferase; NSCLC, non–small‐cell lung cancer; PDAC, pancreatic ductal adenocarcinoma; SASP, senescence‐associated secretory phenotype; Treg, regulatory T cell.

#### 3.1.2 Cytokine and Chemokine Signaling Network

Intratumoral immune dynamics are shaped by soluble mediators that regulate immune‐cell recruitment, activation, and suppression (Figure 2A) [33]. T helper 1 (Th1)‐associated chemokines, including CXCL9 and CXCL10, signal through CXCR3 and are characteristic of inflamed, T‐cell‐rich tumors; their expression has also been associated with response to ICB [34, 83]. IFN‐γ produced by activated lymphocytes reinforces CXCL9/CXCL10–CXCR3 signaling, promoting effector T‐cell recruitment and positioning within tumors [33, 83]. CXCR3 signaling can also promote the accumulation and positioning of CXCR3^+^ Tregs, which can restrict local CD8^+^ T‐cell responses [84]. Suppressive cytokines act through distinct mechanisms. Transforming growth factor‐β (TGF‐β) suppresses T‐cell activity while promoting fibroblast activation and ECM remodeling, thereby reinforcing immune exclusion. Interleukin‐10 (IL‐10) can further impair APC function and limit productive T‐cell responses in some tumor settings [35, 46, 85]. Chemokine‐driven recruitment is therefore not inherently beneficial; its effect depends on which cells are recruited and the suppressive signals they encounter [86]. This has motivated strategies that block suppressive pathways such as TGF‐β signaling or enhance chemokine axes that favor effector‐cell recruitment [33].

#### 3.1.3 Metabolic Checkpoints: The Hypoxia–Acidity–Redox Triad

Poor perfusion creates hypoxic and nutrient‐restricted niches that impose metabolic stress on infiltrating immune cells, limiting effector function and promoting suppressive cell states (Figure 2A) [36]. A central mediator of this response is hypoxia‐inducible factor 1α (HIF‐1α), which coordinates adaptation to low oxygen in both malignant and immune cells [7]. Its activation can increase immunoregulatory molecules such as PD‐L1, reshape myeloid‐cell function, and amplify extracellular adenosine signaling through CD39/CD73 and adenosine receptors [87, 88]. Together, these changes suppress intratumoral T‐cell proliferation and effector function and can weaken local immune control [89, 90]. Hypoxia can also reduce tumor‐antigen visibility through autophagy‐dependent degradation of MHC I, while the accompanying accumulation of lactate and extracellular acidification further impairs DC and cytotoxic lymphocyte function [44, 89]. Redox stress adds a distinct, bidirectional component. Reactive oxygen species (ROS) can impair lymphocyte survival and function, but can also promote oxidative damage in tumor cells, including ferroptotic cell death [91]. IFN‐γ released by activated CD8^+^ T cells can suppress tumor‐cell SLC3A2/SLC7A11 expression, reduce cystine uptake, and increase susceptibility to ferroptosis [92]. Together, hypoxia, extracellular acidity, and redox imbalance impose interacting metabolic constraints on antitumor immunity and provide complementary, rather than unitary, therapeutic targets [87].

#### 3.1.4 Stromal and Vascular Immune Gating Axis

Stromal and vascular compartments jointly determine whether immune cells can enter tumors and reach malignant nests (Figure 2A) [93, 94]. Cancer‐associated fibroblasts (CAFs) can create a restrictive stromal landscape through ECM deposition and TGF‐β‐associated remodeling. In collagen‐rich or fibroblast‐dense regions, T‐cell movement is slowed and contact with tumor cells can be reduced. CAF‐derived signals can impose an additional functional constraint on infiltrating lymphocytes [37, 61]. This stromal control is not confined to fibroblasts. Adipocytes and mesenchymal stromal cells can alter matrix composition, metabolite availability, and local cytokine signaling, adding further heterogeneity to the tissue environment encountered by immune cells [95, 96].

The vasculature controls an earlier step. VEGF‐driven angiogenesis produces disorganized, poorly perfused vessels, and tumor endothelial cells can impair leukocyte adhesion and extravasation [97, 98, 99]. Poor perfusion then deepens hypoxia, linking defective entry to reduced immune‐cell fitness within the tumor [97, 98, 99]. A related principle operates in hematologic malignancies, where specialized bone‐marrow vascular niches support leukemic stem‐cell maintenance and treatment resistance [96, 100, 101]. Immune exclusion can therefore develop at successive spatial checkpoints. Cells may fail to cross the vascular barrier, traverse the stroma, or remain functional after entry [93]. This distinction matters therapeutically. Restoring immune access may require vascular normalization, stromal remodeling, or both, particularly when immunotherapy depends on sustained contact between effector cells and malignant cells [102].

### 3.2 Cell‐State and Tumor‐Intrinsic Regulators of TME Immune Set‐Points

#### 3.2.1 Epigenetic Programming and Senescence‐Associated Secretory Phenotype

Epigenetic remodeling and therapy‐induced senescence can both prolong immune dysfunction beyond the signals that initiated it, but through distinct mechanisms. Microenvironmental signals can establish gene‐regulatory states that persist beyond the initiating stimulus in both tumor and immune cells (Figure 2A) [43, 103, 104]. Tumor cells may epigenetically silence antigen‐presentation and T‐cell‐recruitment programs, and exhausted T cells can retain chromatin configurations that limit sustained functional recovery [43]. These persistent states may outlast the signals that induced them, limiting full recovery of antitumor immunity. Epigenetic therapies offer one route to reversing this memory. In selected tumor models, inhibition of DNA methyltransferases or histone deacetylases reactivates IFN‐responsive and antigen‐presentation programs and increases chemokine expression, improving tumor immunogenicity and responsiveness to ICB [105, 106, 107].

Therapy‐induced senescence operates through a different route [59]. Senescent tumor and stromal cells may develop a senescence‐associated secretory phenotype (SASP) composed of cytokines, chemokines, growth factors, and matrix‐remodeling mediators. Its effects depend on composition and context. Some SASP programs support immune clearance of senescent cells. Others sustain chronic inflammation, recruit suppressive myeloid populations, or facilitate tumor regrowth [59, 60]. Persistent senescent cells can therefore preserve a treatment‐altered microenvironment long after the initial insult. This has prompted interest in senolytic strategies and selective modulation of SASP components, although when and in which tumors these approaches will improve antitumor immunity remains uncertain [59].

#### 3.2.2 Tumor‐Intrinsic Oncogenic Programs

Oncogenic pathways can program TME immune phenotypes through several processes, including antigen visibility, chemokine production, and myeloid‐cell recruitment (Figure 2A) [108, 109]. For example, Wnt/β‐catenin signaling limits DC recruitment and drives T‐cell‐poor tumors [110, 111]. Constitutive STAT3 signaling can suppress proinflammatory chemokines and favor suppressive myeloid accumulation in some tumor models. PTEN loss can impair T‐cell priming and promote resistance to T‐cell‐mediated killing through distinct mechanisms [108, 109]. Many of these oncogenic programs also intersect with IFN signaling and innate immune sensing, including the cGAS–STING pathway, with downstream effects on DC activation, antigen presentation, and T‐cell priming [112, 113]. Because tumor‐intrinsic states can vary across tumor regions, their immune effects may also be spatially heterogeneous, contributing to regional immune escape and heterogeneous therapeutic responses [114].

### 3.3 Distal Systemic Modifiers of TME Immune Set‐Points

#### 3.3.1 Microbiome as a Distal Immune Regulator

The gut microbiome can modify systemic immune homeostasis and, in turn, influence local myeloid–T‐cell interactions within tumors (Figure 2A) [115, 116]. Mechanistically, microbial metabolites and microbial products sensed by pattern‐recognition receptors can modulate DC function, myeloid suppressive programs, and effector‐Treg balance, thereby influencing antigen presentation, inflammatory signaling, and immune‐cell metabolism [117, 118, 119]. Clinically, dysbiosis and antibiotic exposure are associated with impaired ICB responses, motivating investigation of interventions ranging from dietary modulation to defined consortia and fecal microbiota transplantation [120]. Causal relationships remain incompletely defined, and the magnitude and direction of microbiome effects vary by tumor type and between gut and intratumoral communities; these microbial ecosystems should therefore be framed as response modifiers, not universal drivers [121].

### 3.4 Case Node: FOSL2 Links Local Stress Cues to Context‐Dependent TME States

FOSL2/Fra‐2 illustrates how the same transcriptional regulator can support different cellular programs across the TME (Figure 2B). Rather than functioning as a uniform immune suppressor or pan‐cancer driver, FOSL2 activity appears to depend on cell lineage, AP‐1 dimer composition, chromatin accessibility, upstream signaling, and the local microenvironment [20]. Accordingly, its biological effects cannot be inferred from expression alone or readily extrapolated across tumor types and cellular compartments [20]. Similar context‐dependent functions have also been reported in autoimmune and fibroproliferative disorders, where FOSL2 regulates inflammatory and tissue‐remodeling programs [122, 123, 124].

Evidence from individual tumor models illustrates this heterogeneity. In pancreatic ductal adenocarcinoma (PDAC), KRAS‐driven FOSL2 induces CCL28 and promotes Treg recruitment, whereas current evidence does not support a general FOSL2‐dependent chemokine switch involving coordinated suppression of CXCL9/CXCL10 [30, 125]. In glioblastoma, hypoxia‐associated HIF‐1α–FOSL2 signaling induces ANXA1 in tumor cells, promoting the recruitment and polarization of bone‐marrow‐derived macrophages through FPR1/3 [78]. In therapy‐resistant non–small‐cell lung cancer (NSCLC), FOSL2 has been linked to ferroptosis resistance through the NAMPT axis, although whether this mechanism influences neoantigen selection or T‐cell‐mediated killing remains unknown [126]. These examples indicate that FOSL2 does not impose a common immune phenotype across cancers, but instead participates in distinct regulatory circuits defined by tumor lineage and microenvironmental stress.

The same caution applies to chromatin and immune‐regulatory mechanisms. FOSL2‐dependent changes in chromatin accessibility at inflammatory loci have been demonstrated in nontumor systems, but their relevance to tumor immunity requires direct validation [127, 128]. Likewise, although AP‐1 activity intersects broadly with IFN signaling and antigen‐presentation pathways, evidence that FOSL2 itself controls MHC Class I expression across tumor contexts remains limited [32, 129]. Establishing such relationships will require FOSL2‐directed perturbation combined with chromatin profiling, spatial analysis, and functional assessment of immune‐cell responses. Thus, FOSL2 is most informative not as a universal therapeutic target, but as a model for how transcription‐factor function emerges from the interaction between cell identity, signaling context, and tissue environment.

## 4 T‐Cell Dynamics in the TME

The antitumor activity of T cells depends not only on their abundance but also on the functional states they acquire within the TME [130]. Transcriptional, epigenetic, and metabolic regulation influences T‐cell persistence, effector function, and interactions with stromal and myeloid cells [51, 131]. In this context, the efficacy of ICB and adoptive cell therapies depends in part on the presence of T‐cell populations that can enter tumors, persist, and maintain or recover effector function (Figure 3) [132].

**FIGURE 3:**
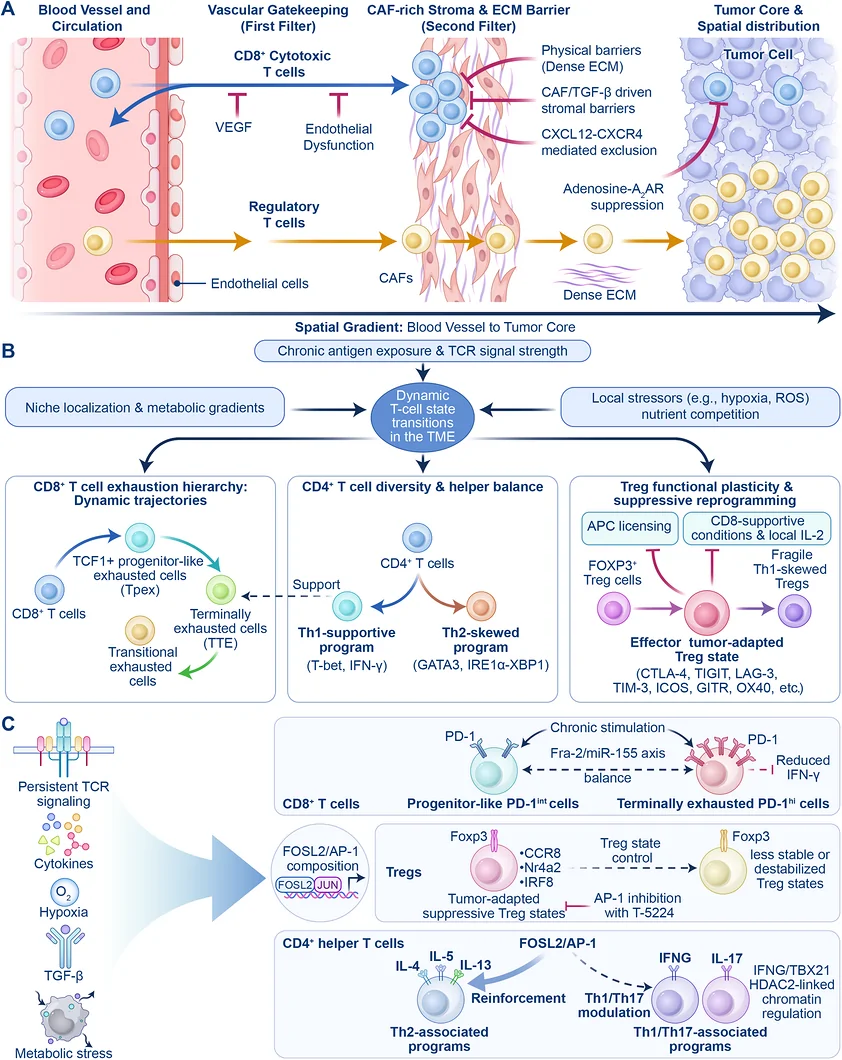
Spatial positioning and state transitions of T cells in the tumor microenvironment. (A) T‐cell entry into tumor‐cell‐rich regions is controlled by sequential vascular, stromal, and biochemical barriers. Vascular endothelial growth factor (VEGF)‐associated endothelial dysfunction, cancer‐associated fibroblast (CAF)‐rich stroma, dense extracellular matrix (ECM), transforming growth factor‐β (TGF‐β)‐driven stromal barriers, CXCL12‐CXCR4‐mediated exclusion, and adenosine‐A2A receptor signaling restrict CD8^+^ T‐cell access while favoring regulatory T‐cell (Treg) accumulation in suppressive niches. (B) Chronic antigen exposure, T‐cell receptor (TCR) signal strength, niche localization, and metabolic stress shape dynamic T‐cell state transitions. CD8^+^ T cells progress from T‐cell factor 1 (TCF1)^+^ progenitor‐like exhausted cells toward transitional and terminally exhausted states, whereas CD4^+^ helper programs and FOXP3^+^ Treg plasticity define the local helper‐suppressive balance. (C) FOSL2/activator protein 1 (AP‐1) is a candidate context‐dependent tuning module for T‐cell states. It is linked to CD8^+^ exhaustion‐associated programs, tumor‐adapted Treg states, and CD4^+^ helper‐cell polarization but should not be interpreted as a master regulator without lineage‐specific functional validation.

### 4.1 Spatial Control of T‐Cell Recruitment and Niche Localization

Effective antitumor immunity requires not only T‐cell recruitment but also access to tumor‐cell regions. T‐cell exclusion can arise at multiple spatial checkpoints, including defective recruitment, impaired extravasation, and stromal barriers that restrict migration into tumor‐cell regions, reducing direct contact between cytotoxic T lymphocytes and malignant cells [133, 134].

#### 4.1.1 Chemokine Axes and Vascular Control of T‐Cell Entry

Chemokine receptor–ligand networks help determine which T‐cell subsets are recruited into tumors and where they accumulate (Figure 3A) [133, 134, 135]. Tumor‐infiltrating effector regulatory T cells (eTregs) commonly use the CCL17/CCL22–CCR4 and CCL1–CCR8 axes for tumor accumulation [136, 137, 138, 139, 140, 141, 142, 143]. Hypoxia can promote CCL28‐dependent recruitment of CCR10+ Tregs [144]. The CXCL9/CXCL10/CXCL11–CXCR3 axis is also not confined to effector‐cell recruitment. Although this pathway supports CTL trafficking, CXCR3 can also promote the intratumoral accumulation and positioning of Tregs, thereby restricting local CD8^+^ T‐cell responses [33, 84, 145, 146, 147]. T‐cell accumulation is also influenced by sphingosine‐1‐phosphate signaling and vascular function. Sphingosine‐1‐phosphate receptor 1 (S1PR1) signaling can promote intratumoral Treg accumulation through STAT3, and endothelial dysfunction can limit immune‐cell extravasation [38, 148, 149]. Tregs can also restrict the development of tumor‐associated high endothelial venules (HEVs), specialized vascular structures that facilitate lymphocyte entry into tumors [39, 150]. VEGF–VEGFR2 signaling can support eTreg accumulation and contribute to vascular abnormalities that limit CD8^+^ T‐cell infiltration [151, 152, 153]. Together, these observations indicate that recruitment and parenchymal access are distinct but related control points in T‐cell positioning within tumors.

#### 4.1.2 ECM Architecture and Active Exclusion

CAF‐rich stroma and a dense ECM can impede CTL migration and favor T‐cell accumulation at the tumor margin [52, 148, 154]. Treg‐CAF crosstalk, including TGF‐β signaling, can reinforce fibroblast activation and ECM deposition, while CAF‐derived CXCL12 can restrict T‐cell access through CXCR4 [155, 156, 157]. Adenosine‐A2AR signaling can additionally suppress T‐cell function after cells enter the tumor (Figure 3A) [53, 158]. A key unresolved question is the extent to which spatial exclusion results from physical restriction or active chemorepulsive signals. In triple‐negative breast cancer (TNBC), quantitative modeling showed that a model incorporating a hypothetical T‐cell chemorepellent more closely reproduced observed CD8^+^ T‐cell spatial profiles than models based on physical restriction alone [40, 159, 160, 161]. The underlying repulsive signals remain unidentified and require experimental validation. Distinguishing physical restriction from active chemorepulsion is therapeutically relevant because physical barriers may require stromal or matrix remodeling, while active exclusion would require identification and blockade of the responsible signals.

#### 4.1.3 TLSs and Tumor‐Draining Lymph Nodes as T‐Cell Niches

TLSs and tumor‐draining lymph nodes (tdLNs) are specialized lymphoid niches that sustain T‐cell priming and progenitor states, but local regulatory populations can also restrain these functions. tdLNs provide sites for tumor‐specific T‐cell priming and maintenance, while TLSs organize related immune interactions within or adjacent to tumors [162, 163]. TLSs resemble secondary lymphoid organs, with segregated T‐cell zones and B‐cell follicles assembled through inflammatory cues and stromal chemokines such as CXCL13. Their presence is often associated with stronger local antitumor immunity [164, 165]. TCF1^+^ progenitor‐exhausted CD8^+^ T cells (Tpex) are enriched in tdLNs and tumor‐associated lymphoid niches, including TLSs. Within these niches, they are often found in proximity to DCs [54, 140]. These cells can retain self‐renewal capacity and generate more differentiated effector progeny. However, direct evidence that TLS architecture is required to maintain TCF1^+^ progenitor states remain limited, and the extent of this niche dependence may vary across tumor contexts.

Within TLS and tdLN niches, the quality of T‐cell‐antigen‐presenting cell (APC) interactions is shaped by local regulatory populations. T follicular helper (Tfh) cells contribute to TLS organization, in part through CXCL13. T follicular regulatory (Tfr) cells can restrain follicular responses. In human breast cancer, Tfr‐mediated suppression has been linked to a glycoprotein A repetitions predominant (GARP)‐associated TGF‐β‐dependent mechanism [165, 166]. Conventional Tregs provide a separate layer of suppression through CTLA‐4, which removes CD80 and CD86 from APCs through trans‐endocytosis, reducing CD28‐dependent costimulation and limiting local T‐cell priming and expansion [167, 168, 169]. Supportive lymphoid niches are therefore shaped by both signals that sustain T‐cell responses and regulatory populations that restrain them.

### 4.2 Lineage Commitment: Differentiation Trajectories and Subtype Heterogeneity

The spatial distribution and niche localization of T cells can influence their differentiation and functional fate within the TME (Figure 3B). Chronic antigen exposure and local stressors drive T‐cell along distinct differentiation trajectories, with consequences for effector function, persistence, and treatment response [170, 171]. Single‐cell and other high‐dimensional approaches have therefore shifted the field from fixed subset labels toward dynamic, hierarchically related T‐cell states [170, 171]. These states are maintained by interacting transcriptional, epigenetic, and metabolic programs [170, 171].

#### 4.2.1 CD8+ T‐Cell Exhaustion Trajectories: Progenitor Maintenance and Terminal Differentiation

Persistent tumor‐antigen exposure drives CD8^+^ T cells through hierarchically related exhaustion states that can follow divergent differentiation trajectories [172]. Among these, the TCF1^+^ progenitor‐like compartment (Tpex) retains self‐renewal capacity and preferentially sustains responses to ICB. More differentiated terminally exhausted cells lose TCF1 expression, upregulate inhibitory receptors such as TIM‐3, LAG‐3, and TIGIT together with CD39, and are less responsive to checkpoint‐mediated reinvigoration [131, 171, 173, 174, 175]. An exhausted phenotype does not necessarily indicate complete functional loss. In some settings, terminally exhausted cells retain cytotoxic capacity despite high inhibitory‐receptor expression [173]. Thymocyte selection‐associated high mobility group box (TOX) is a central regulator of exhaustion differentiation, not a simple marker of irreversible functional failure [55, 176].

#### 4.2.2 CD4+ T‐Cell Diversity and the Helper‐Suppressive Balance

CD4^+^ T‐cell lineages regulate both cytotoxic and humoral antitumor immunity through diverse helper and suppressive functions [174, 177]. Th1 differentiation is characterized by T‐bet expression [178], and Th1 support antigen presentation and CD8^+^ effector maintenance through IFN‐γ‐ and TNF‐α‐dominant circuits and are often associated with favorable outcomes [177, 179]. Th2 polarization is characterized by GATA3 and can be supported by the IRE1α‐XBP1 stress‐response pathway. Although Type 2 inflammation is often less supportive of classical CTL‐dominant immunity, Th2 cells can exert antitumor effects in selected contexts [180]. When tumors evade CD8^+^ T‐cell recognition through MHC‐I loss, CD4‐dependent effector mechanisms may become increasingly important. More broadly, these observations argue against a simple Th1‐antitumor/Th2‐protumor classification of CD4^+^ T‐cell states [181].

#### 4.2.3 Treg Functional Plasticity and Suppressive Reprogramming

Tumor‐infiltrating FOXP3^+^ Tregs commonly acquire activated effector‐like phenotypes [9, 182]. Persistent TCR stimulation and cytokine exposure can drive an activated eTreg phenotype, marked by increased expression of multiple inhibitory and costimulatory receptors together with high CD25 expression (Figure 3C) [183, 184]. These features support suppression through complementary mechanisms, including CTLA‐4‐mediated inhibition of APC costimulation and high‐affinity IL‐2 consumption through CD25 [56, 185].

Although developmental origin can sometimes be approximated using markers such as Helios and NRP1, neither provides an absolute distinction between thymus‐derived and peripherally induced Tregs. Local tissue signals can subsequently modify Treg phenotype after tumor entry [168, 186]. Tumor‐enriched features further tune Treg fitness and retention within tissues. LAYN marks highly activated tumor‐infiltrating Treg states [187, 188]. CD74 supports tumor Treg accumulation and Foxp3 stability, in part through effects on cytoskeletal organization [168, 189].

FOXP3 operates within a broader transcriptional network that sustains Treg identity and function. In tumor Tregs, nuclear factor of activated T cells (NFAT) and AP‐1 family factors, including BATF, contribute to effector and suppressive transcriptional programs [190]. Metabolic adaptation further maintains Treg fitness, with lactate and lipid metabolism supporting survival and suppressive activity in nutrient‐poor tumors [191]. This functional state is not irreversible. Fragile Tregs retain FOXP3 expression but show reduced suppressive activity under inflammatory conditions [192]. In a melanoma model responsive to PD‐1 blockade, intratumoral Tregs acquired T‐bet and IFN‐γ expression and a Th1‐like phenotype, allowing CD8^+^ T cells to escape Treg‐mediated suppression [193].

### 4.3 Molecular Engines of Dysfunction in Effector T Cells

Effector T‐cell dysfunction becomes progressively stabilized through cell‐intrinsic transcriptional, epigenetic, and metabolic programs [57, 194, 195]. Chronic antigen stimulation, metabolic stress, and spatial restriction reinforce these programs and limit functional recovery, although some features remain reversible.

#### 4.3.1 Transcriptional Programs Governing T‐Cell Dysfunction

Prolonged TCR signaling, together with metabolic and cytokine stress, engages transcriptional programs that maintain T‐cell dysfunction [196, 197, 198]. Sustained NFAT activity, particularly when uncoupled from adequate AP‐1 cooperation, induces TOX and NR4A family factors and promotes inhibitory‐receptor expression [54, 131]. Other transcriptional circuits preserve less differentiated states. TCF1 and BACH2 instead preserve less differentiated exhausted states by maintaining progenitor features and restraining terminal differentiation [131, 140]. Differences in T‐bet and EOMES regulation across tumors and chronic infection models suggest that T‐cell exhaustion is built on a shared regulatory framework but shaped by the local tissue environment [51, 57].

#### 4.3.2 Metabolic Repression and Mitochondrial Insufficiency

Metabolic failure is a major mechanism sustaining effector T‐cell dysfunction. Hypoxia, nutrient deprivation, acidosis, and inhibitory signaling restrict energy production and undermine mitochondrial fitness [82, 194, 199, 200]. Activated effector T cells (Teffs) rely heavily on glycolysis to support proliferation and cytotoxic activity, but glucose limitation and inhibitory‐receptor signaling constrain this metabolic program [51, 82]. PD‐1 signaling can further reprogram T‐cell metabolism by suppressing glycolysis while favoring lipolysis and fatty‐acid oxidation [51, 201]. Mitochondrial dysfunction represents another prominent feature of dysfunctional CD8^+^ TILs, with reduced respiratory capacity, altered membrane potential, and increased oxidative stress limiting metabolic fitness and effector function [195]. Acidosis and lactate accumulation impose an additional metabolic burden by suppressing effector activity and disrupting mitochondrial function [51, 195]. Importantly, transcriptional signatures of glycolysis or lipid metabolism do not necessarily reflect metabolic flux; mechanistic conclusions therefore require functional or perturbation‐based validation [54, 195].

#### 4.3.3 Epigenetic Stabilization of Dysfunctional States

Progressive loss of epigenetic plasticity limits the reversibility of T‐cell exhaustion [51, 131]. Chronic antigen exposure induces DNMT3A‐dependent de novo DNA‐methylation programs that reinforce T‐cell exhaustion and restrict functional plasticity [202]. TET2 also influences CD8^+^ T‐cell fate: its loss promotes memory differentiation during acute responses, whereas under chronic stimulation TET2 facilitates progression from stem‐like exhausted progenitors toward more differentiated exhausted states [203, 204]. Exhausted T cells retain a durable epigenetic scar, with incomplete recovery of memory‐associated chromatin programs even after chronic antigen is removed [205]. PD‐1 blockade can restore transcriptional activity without extensively remodeling this underlying epigenetic architecture, limiting the durability of T‐cell reinvigoration [57, 206]. Progenitor‐like exhausted T cells retain greater responsiveness to PD‐1 blockade, whereas terminally exhausted populations are less readily reinvigorated [57, 140]. Thus, inhibitory‐receptor expression alone does not define reversibility; the underlying epigenetic state also constrains whether dysfunctional T cells remain amenable to therapeutic reinvigoration.

#### 4.3.4 Intercellular Suppression and Resource Competition

Cell‐extrinsic mechanisms within the TME restrict effector T‐cell activation, persistence, and access to essential costimulatory and growth signals [207]. Tregs limit costimulation through CTLA‐4‐mediated removal of CD80 and CD86 from APCs, including through trans‐endocytosis [208]. This reduces CD28 signaling in effector T cells [208]. They also use IL‐10, TGF‐β, and IL‐35 to suppress APC function and effector T‐cell responses through distinct, context‐dependent mechanisms [209]. Resource competition adds another layer of suppression. High CD25 expression can give Tregs preferential access to IL‐2 and, in some settings, restrict its availability to effector T cells, whereas CD39/CD73‐dependent adenosine generation suppresses T‐cell activation [58, 210]. The persistence of this suppressive compartment is supported by metabolic adaptation, with tumor Tregs using oxidative phosphorylation and lipid metabolic programs in selected nutrient‐stressed contexts [211]. Together, these mechanisms suppress effector T‐cell activation and persistence through complementary cell‐extrinsic pathways [212].

### 4.4 Case Node: FOSL2/AP‐1 as a Context‐Dependent Modulator of T‐Cell State Transitions

Fra‐2, encoded by *FOSL2*, is a context‐dependent AP‐1 component whose effects on T‐cell differentiation vary with lineage, activation state, and partner composition (Figure 3C). Its function is therefore better viewed as modulation of state‐specific transcriptional programs than as a uniform lineage switch [26, 213, 214, 215, 216].

In chronic viral infection, Fosl2 has been implicated in the differentiation of exhausted CD8^+^ T‐cell subsets. Fosl2 expression is higher in PD‐1^int^ progenitor than in PD‐1^hi^ terminally exhausted cells, and enforced Fosl2 expression counteracts miR‐155‐driven features of terminal exhaustion [217, 218, 219]. CTLA‐4 signaling can also reduce Fra‐2 phosphorylation during CD8^+^ T‐cell differentiation, indicating that Fra‐2 activity is sensitive to coinhibitory signaling [220].

Fra‐2 also influences regulatory and helper T‐cell differentiation. In nontumor settings, increased *Fosl2* activity can impair Treg development by repressing *Foxp3* and other genes associated with Treg differentiation [213, 219, 221, 222]. In tumor‐infiltrating Tregs, AP‐1 activity contributes to activation‐associated transcriptional and chromatin programs, but a specific requirement for Fra‐2 has not been established [213, 219, 221, 222]. Pharmacologic inhibition of c‐Fos/AP‐1 with T‐5224 can reduce FoxP3 and PD‐1 induction in specific Treg differentiation settings, further supporting a role for AP‐1 family activity but not a FOSL2‐specific requirement [222]. In CD4^+^ helper T cells, Fra‐2 has been linked to Th2 polarization and to regulation of Th17 differentiation and lineage stability, with its effects varying across experimental systems and AP‐1 partner composition [26, 214, 216, 219, 223, 224].

Together, these findings indicate that Fra‐2 participates in T‐cell differentiation and adaptation in a state‐ and lineage‐dependent manner but does not support its classification as a master regulator of exhaustion, Treg stability, or helper‐cell polarization. Within tumors, FOSL2 expression or AP‐1 motif activity should therefore be treated as evidence of a candidate regulatory state rather than proof that Fra‐2 is the causal driver. Lineage‐specific *FOSL2* perturbation combined with chromatin profiling and functional immune assays will be required to determine whether Fra‐2 directly controls T‐cell dysfunction or plasticity in the TME.

## 5 B‐Cell Organization and Functional Plasticity in Tumors

Tumor‐infiltrating B cells occupy a functional spectrum shaped by their spatial organization and differentiation state. Within mature TLSs, B cells can support antitumor immunity through antigen presentation, germinal‐center (GC) reactions, and tumor‐reactive antibody responses [225, 226, 227, 228, 229]. In other microenvironmental contexts, B cells can adopt regulatory phenotypes that suppress effector immunity [225, 226, 227, 228, 229].

### 5.1 Local B‐Cell Maturation and Immune Coordination in TLSs

Mature, GC‐containing TLSs provide local niches for B‐cell activation, somatic hypermutation, class‐switch recombination, and differentiation into memory B cells and plasma cells (Figure 4). GC‐containing TLSs can coordinate local T‐ and B‐cell responses, and mature TLSs have been associated with favorable clinical outcomes and response to ICB across several solid tumors [226, 229, 230]. Less organized lymphoid aggregates lack some features of mature GC reactions, and their relationship with antitumor immunity is more variable. TLS function is also shaped by the surrounding microenvironment, where lymphoid aggregates can coexist with suppressive myeloid or stromal niches [225, 226].

**FIGURE 4:**
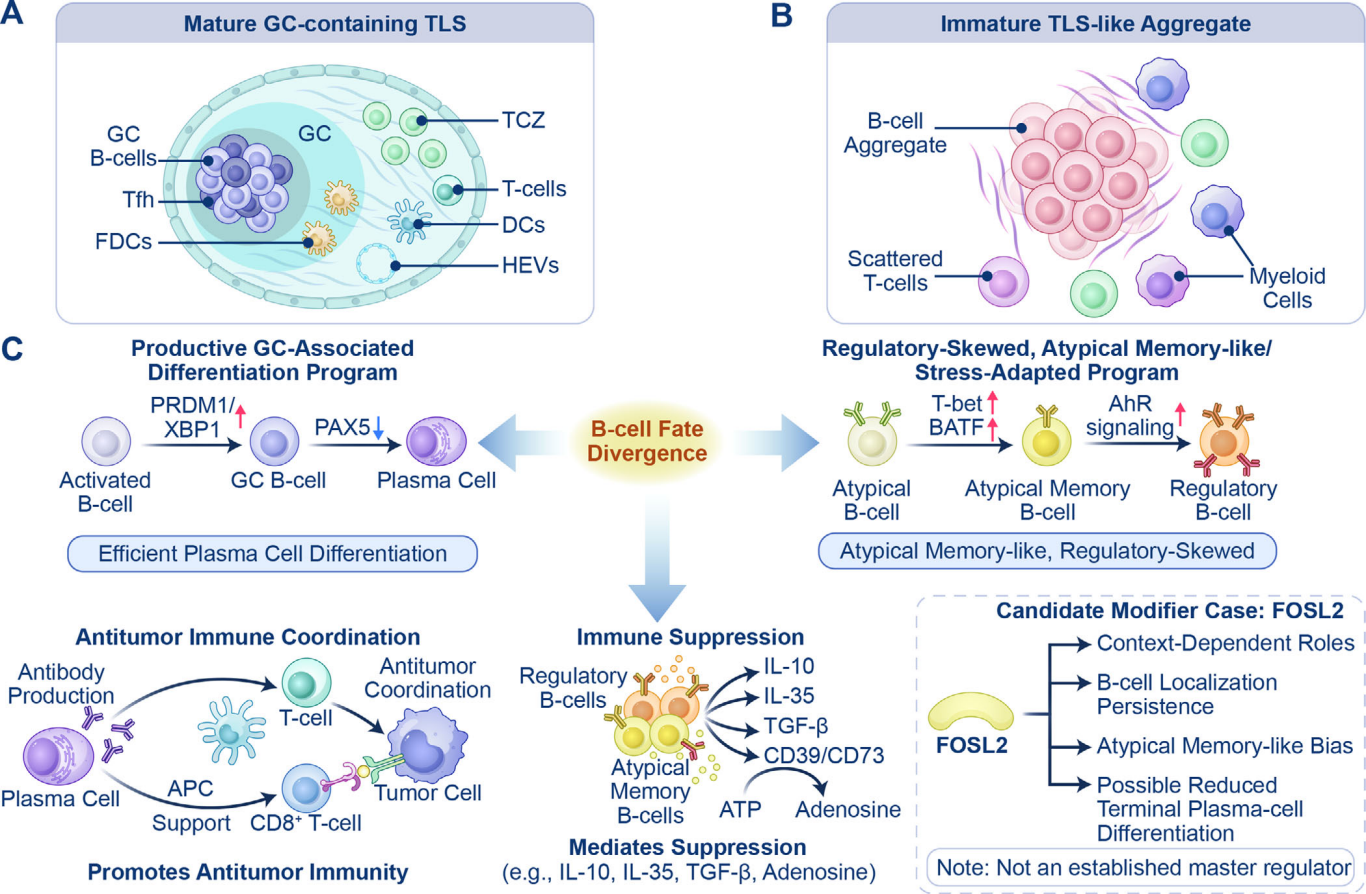
TLS‐dependent B‐cell fate programs in tumors. (A) Schematic representation of a mature germinal center (GC)‐containing tertiary lymphoid structure (TLS). Mature TLSs display organized T–B‐cell compartmentalization and contain GC B cells, T follicular helper (Tfh) cells, follicular dendritic cells (FDCs), dendritic cells (DCs), high endothelial venules (HEVs), and a T‐cell zone (TCZ). This architecture supports productive GC reactions associated with B‐cell activation, somatic hypermutation, class‐switch recombination, and plasma‐cell differentiation. (B) Immature TLS‐like aggregate lack a productive GC structure. B‐cell aggregates coexist with scattered T cells and myeloid cells but lack clear spatial segregation, potentially limiting the coordinated humoral and cellular immune responses. (C) Proposed B‐cell fate trajectories linked to TLS maturation. In mature GC‐containing TLSs, GC‐experienced B cells may undergo productive differentiation toward antibody‐secreting plasma cells, supported by PRDM1/BLIMP1 and XBP1. Under chronic inflammatory, metabolic, or antigenic stress, B cells may acquire atypical memory‐like, stress‐adapted, or regulatory features associated with T‐bet, BATF, aryl hydrocarbon receptor (AhR) signaling, and immunosuppressive mediators. FOSL2/activator protein 1 (AP‐1) is proposed as a context‐dependent modifier of B‐cell state transitions, although its direct role remains to be functionally established. AhR, aryl hydrocarbon receptor; APC, antigen‐presenting cell; AP‐1, activator protein 1; ATP, adenosine triphosphate; DC, dendritic cell; FDC, follicular dendritic cell; GC, germinal center; HEV, high endothelial venule; IL, interleukin; TCZ, T‐cell zone; Tfh, T follicular helper; TGF‐β, transforming growth factor‐β; TLS, tertiary lymphoid structure.

B cells within TLSs function as local APCs and support CD4^+^ T‐cell activation [231]. Tumor‐infiltrating plasma cells can also generate antigen‐specific antibodies. In human papillomavirus (HPV)‐positive head and neck cancer, intratumoral B‐lineage cells produce antibodies against HPV E2, E6, and E7, whereas clonally expanded antibody‐secreting cells in high‐grade serous ovarian cancer generate tumor‐reactive antibodies, including antibodies targeting MMP14 [232, 233, 234]. The consequences of these local antibody responses depend on antigen specificity, isotype, and tumor context and cannot be predicted from antibody abundance alone. B‐cell receptor clonality and somatic hypermutation can indicate antigen‐driven selection, but establishing tumor‐antigen specificity requires direct antigen‐binding and functional analyses [235, 236, 237].

### 5.2 B‐Cell Differentiation Trajectories and Regulatory Diversion

Single‐cell and spatial studies reveal substantial heterogeneity among tumor‐infiltrating B cells, with differentiation state closely linked to local immune function [227, 238]. GC‐associated maturation can give rise to memory B‐cell and plasma‐cell responses, with plasma‐cell commitment marked by induction of PRDM1 (encoding BLIMP‐1), repression of PAX5, and activation of XBP1‐dependent secretory programs [227, 239]. GC‐associated B cells can support antigen presentation and T‐cell coordination. Differentiated plasma cells provide a local source of antibody production [230, 240, 241].

Tumor‐derived and chronic inflammatory signals can also favor extrafollicular or regulatory B‐cell states. Atypical‐memory B cells frequently express T‐bet and can acquire immunoregulatory features in selected tumor contexts. In hepatocellular carcinoma, nonswitched memory B cells with a TBET^+^CD11c^+^ phenotype express PD‐L1, CD25, and granzyme B, and tumor‐exposed B cells can acquire suppressive activity that reduces CD8^+^ T‐cell costimulation [227, 238]. Breg‐like populations use diverse mechanisms to restrain antitumor immunity, including IL‐10, IL‐35, TGF‐β, PD‐L1, and CD39/CD73‐dependent adenosinergic signaling [230, 242]. Additional suppressive phenotypes include PD‐L1^+^ B cells and granzyme B‐expressing B cells; in IL‐21‐rich settings, the latter can directly inhibit T‐cell proliferation through granzyme B‐dependent mechanisms [230, 240, 241, 243]. Together, these findings argue that B‐cell function in tumors is shaped by local architecture and differentiation trajectory, not by B‐cell abundance alone.

### 5.3 Case Node: FOSL2/AP‐1 as a Candidate Regulator of B‐Cell State Transitions

Fra‐2 has an established role in B‐cell development and differentiation outside the tumor setting (Figure 4). B‐cell‐specific loss of Fra‐2 impairs proliferation and differentiation, in part through reduced FOXO1‐ and IRF4‐dependent transcriptional programs [25, 244]. Whether this regulatory circuitry operates in tumor‐infiltrating B cells or contributes to TLS maturation remains unknown. Pan‐cancer single‐cell analyses have identified Fra‐2 as a candidate transcriptional regulator associated with atypical‐memory intratumoral B‐cell states, but whether FOSL2 promotes, maintains, or merely marks these states remains unknown [25, 245]. One possibility is that altered AP‐1 activity contributes to the balance among GC, atypical memory, and antibody‐secreting‐cell differentiation, but this has not been demonstrated specifically for FOSL2. Determining whether FOSL2 directly regulates tumor‐associated B‐cell differentiation will require B‐cell‐specific perturbation coupled with lineage tracing, chromatin profiling, and spatial analysis of TLS organization.

## 6 Innate and Innate‐Like Lymphocytes

This section focuses on NK and iNKT cells and the mechanisms that regulate their tumor access, effector function, metabolic fitness, and phenotypic plasticity. We also discuss engineered iNKT platforms and the lineage‐dependent roles of FOSL2/Fra‐2 and AP‐1 in innate lymphocyte differentiation and function.

### 6.1 Spatial Exclusion, Dysfunction, and ILC1‐Like Reprogramming of NK Cells

Tumors disable NK‐cell surveillance through a combination of spatial exclusion, functional suppression, and phenotypic reprogramming. NK cells are cytotoxic group 1 innate lymphoid cells. ILC1s are generally more tissue resident and display distinct cytotoxic and cytokine‐producing properties. Tumor‐associated signals, particularly TGF‐β, can drive NK cells toward ILC1‐like states with reduced cytotoxic capacity and increased tissue‐residency features (Figure 5) [246].

**FIGURE 5:**
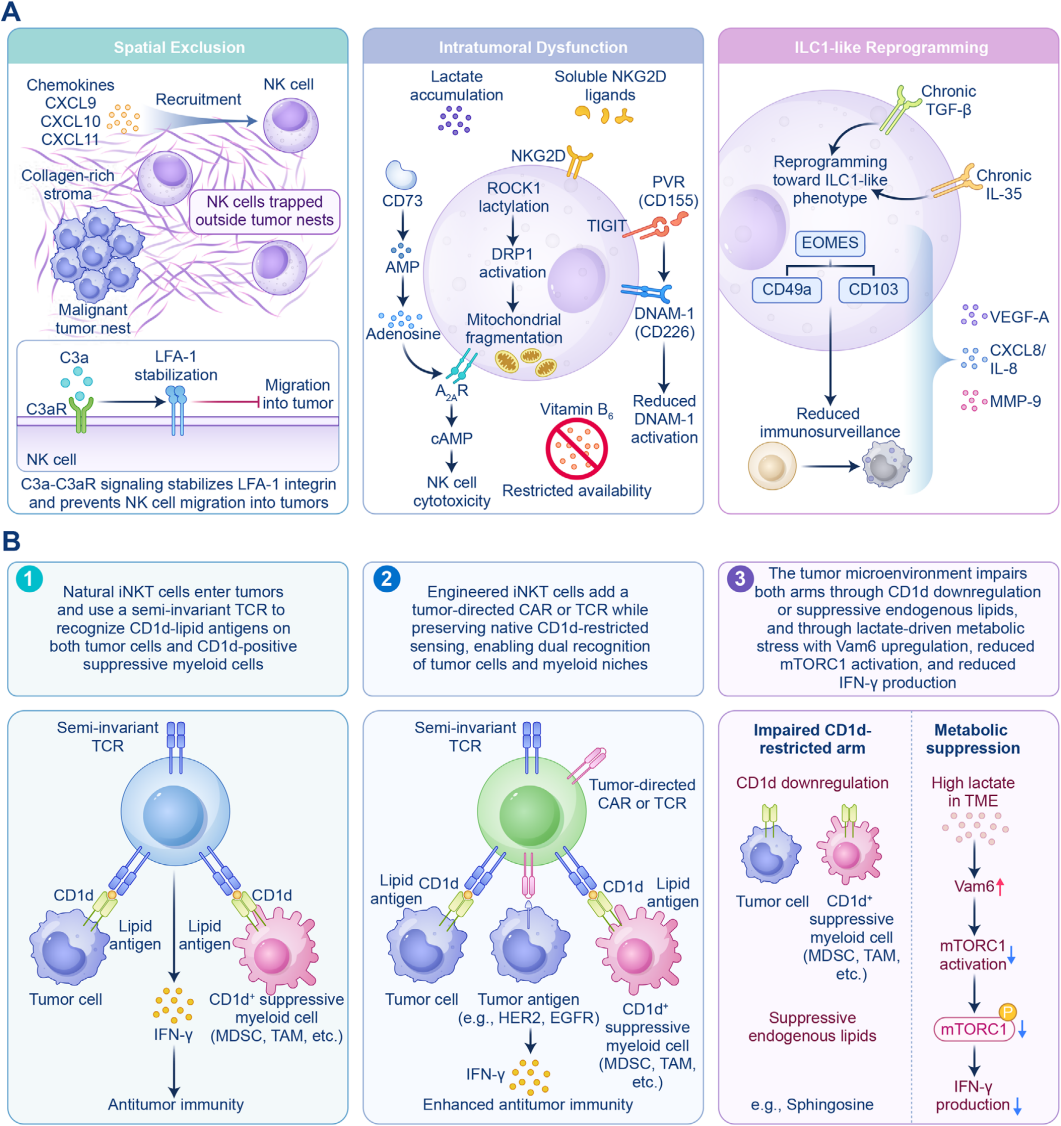
Spatial restriction and dysfunction of NK‐cell and iNKT cell immunity in tumors. (A) Natural killer (NK) cell antitumor activity is progressively constrained at three levels in the tumor microenvironment. (1) Spatial exclusion: NK cells recruited by CXCL9, CXCL10, and CXCL11 can be trapped outside malignant nests through C3a–C3aR‐mediated stabilization of high‐affinity LFA‐1, which promotes stromal adhesion and limits migration into tumor‐cell‐rich regions. (2) Functional erosion: NK cells that enter the tumor bed undergo progressive dysfunction driven by lactate‐induced ROCK1 lactylation, DRP1‐dependent mitochondrial fragmentation, soluble NKG2D ligands, PVR/CD155–TIGIT signaling with reduced DNAM‐1/CD226 activation, CD73‐dependent adenosine–A2A receptor signaling, and nutrient restriction. (3) ILC1‐like reprogramming: Chronic exposure to transforming growth factor‐β (TGF‐β) and, in some contexts, interleukin‐35 (IL‐35) can redirect NK cells toward an innate lymphoid cell Type 1 (ILC1)‐like tissue‐resident state marked by EOMES downregulation and increased CD49a and CD103, with reduced immunosurveillance and, in some settings, increased expression of tissue‐remodeling mediators such as VEGF‐A, CXCL8/IL‐8, and MMP‐9. (B) Invariant natural killer T (iNKT) cells recognize CD1d‐presented lipid antigens through a semi‐invariant T‐cell receptor (TCR), allowing engagement of both tumor cells and CD1d^+^ suppressive myeloid cells, including tumor‐associated macrophages (TAMs) and myeloid‐derived suppressor cells (MDSCs). Engineered iNKT platforms add a tumor‐directed chimeric antigen receptor (CAR) or TCR while preserving native CD1d‐restricted sensing, thereby enabling the dual recognition of malignant cells and suppressive myeloid niches. However, solid tumors can impair both arms through CD1d downregulation, suppressive endogenous lipids, and lactate‐driven metabolic stress, which is associated with increased Vam6 expression, reduced mTORC1 activation, and impaired interferon‐γ (IFN‐γ) production. CAR, chimeric antigen receptor; ILC1, innate lymphoid cell Type 1; iNKT, invariant natural killer T; LFA‐1, lymphocyte function‐associated antigen 1; MDSC, myeloid‐derived suppressor cell; mTORC1, mechanistic target of rapamycin complex 1; NK, natural killer; TAM, tumor‐associated macrophage; TCR, T‐cell receptor.

The first barrier is spatial in nature. CXCR3 ligands, including CXCL9, CXCL10, and CXCL11, recruit NK cells to inflamed tumors; however, recruitment to the tumor bed does not ensure effective intratumoral localization [247, 248, 249]. In mouse some solid tumors, C3a‐C3aR signaling promotes a high‐affinity conformation of LFA‐1 on NK cells, increasing adhesive restraint and reducing NK‐cell infiltration into tumors [250]. NK‐cell dysfunction can also weaken immune coordination because NK‐derived CCL5 and XCL1 promote cDC1 recruitment into tumors; reduced NK‐cell access or function may therefore impair downstream CD8^+^ T‐cell priming [251, 252].

After entry, local pressures rapidly impair NK‐cell effector function [253]. In acute myeloid leukemia, lactate‐induced ROCK1 lactylation activates ROCK1‐DRP1 signaling, promotes mitochondrial fragmentation, and impairs NK‐cell cytotoxicity [254, 255]. Tumors can also reduce NKG2D‐mediated activation through soluble NKG2D ligands, while PVR/CD155 engagement of TIGIT shifts signaling away from the activating receptor DNAM‐1/CD226 [253]. CD73‐driven adenosine‐A2A receptor signaling provides an additional inhibitory pathway, while nutrient competition can further impair NK‐cell function; in PDAC, tumor consumption of vitamin B6 restricts its availability to NK cells and compromises cytotoxicity [88, 256, 257].

With chronic tumor‐associated signaling, functional impairment can be accompanied by phenotypic reprogramming. TGF‐β can promote NK‐to‐ILC1‐like reprogramming characterized by reduced EOMES and acquisition of tissue‐residency markers such as CD49a and CD103 [258, 259]. More recently, prolonged IL‐35 exposure was shown to induce a similar phenotype in human NK cells in vitro through autocrine TGF‐β signaling, although its contribution to NK‐cell conversion within tumors remains to be established in vivo [258, 259]. This shift reduces immunosurveillance and, in some contexts, is accompanied by proangiogenic features, including increased VEGF‐A and CXCL8/IL‐8 production [258, 259]. Together, these mechanisms can limit NK‐cell immunity at multiple stages by restricting tumor access, rapidly impairing effector function after entry, and, in selected settings, promoting tissue‐resident ILC1‐like states with reduced cytotoxicity (Figure 5A).

### 6.2 INKT Cells and Engineered iNKT Platforms

Invariant natural killer T (iNKT) cells are innate‐like T lymphocytes that combine tissue surveillance, rapid cytokine release, and cytotoxic activity (Figure 5B). Their relevance to solid tumor immunotherapy is based on two features. iNKT cells can traffic into tumors through chemokine‐receptor pathways that include CXCR3 and CXCR4, although tumor entry does not ensure effective intratumoral migration, antigen scanning, or activation [260, 261]. Their semi‐invariant TCR recognizes lipid antigens presented by the monomorphic molecule CD1d, enabling direct interactions with CD1d‐expressing malignant and suppressive myeloid cells, including TAMs and MDSCs [260, 262, 263, 264]. Because CD1d expression on malignant cells is variable across solid tumors, recognition of CD1d‐expressing suppressive myeloid populations may provide an additional antitumor mechanism.

Engineered iNKT platforms extend this biology by adding a tumor‐directed CAR or TCR while preserving the endogenous semi‐invariant TCR [260]. The creates the potential for dual recognition: the engineered receptor redirects iNKT cells toward malignant cells, while the endogenous TCR preserves CD1d‐restricted recognition of myeloid populations within the TME. This property is particularly relevant in solid tumors in which malignant cell CD1d expression is limited or heterogeneous.

Solid tumors can compromise iNKT‐cell immunity at both recognition and effector stages. Reduced CD1d expression or altered lipid‐antigen presentation limits endogenous tumor recognition. Metabolic stress acts later, after iNKT cells enter the tumor [265, 266]. In mouse tumor models, tumor‐derived lactic acid increases Vam6 expression in intratumoral iNKT cells, promoting AMPK activation, suppressing mTORC1 signaling, and impairing iNKT‐cell effector function [267]. IFN‐γ production falls, although granzyme B production can remain relatively preserved [263, 268]. These findings suggest that engineered iNKT‐cell therapies may need to address two distinct barriers: tumor recognition and metabolic fitness within the TME. Preserving endogenous CD1d‐restricted sensing may provide an additional advantage.

### 6.3 Case Node: FOSL2/AP‐1 as a Lineage‐Sensitive Regulator of Innate Lymphocyte Differentiation

Studies of iNKT‐ and NK‐cell development reveal markedly different roles for FOSL2/Fra‐2 across innate lymphocyte lineages. In iNKT cells, AP‐1 activity is required for terminal maturation. MED23 deficiency blocks the stage 2‐to‐stage 3 transition and reduces c‐Jun expression, whereas ectopic c‐Jun partially restores maturation [269]. Fra‐2 itself exerts a different effect. Fosl2 deficiency increases c‐Jun expression, iNKT‐cell abundance and cytokine production, indicating that individual AP‐1 components can exert distinct, even opposing, effects within the same differentiation program [270, 271].

In NK cells, Fra‐2 appears to have a different role. In an in vitro differentiation system, integrated transcriptional and chromatin‐accessibility analyses identified FOSL2 and EGR2 as candidate regulators of NK‐cell maturation, and knockdown of either factor impaired human NK cell differentiation [24, 272]. For ILC1s, evidence linking FOSL2 to lineage identity or plasticity remains mainly transcriptional, without direct lineage‐specific perturbation. Taken together, these studies argue for a lineage‐sensitive role of FOSL2 in innate lymphocyte differentiation. Its contribution cannot be inferred from AP‐1 activity alone. Whether FOSL2 directly controls NK‐cell dysfunction or NK‐to‐ILC1‐like reprogramming in tumors remains unresolved.

## 7 Myeloid and Stromal Crosstalk

Myeloid cells acquire their tumor‐associated functions through continuous exchange with malignant, stromal, vascular, and metabolic compartments. These interactions influence immune priming, tissue remodeling, and the persistence of suppressive niches. Here, we examine macrophage–stromal circuits, DC trafficking and priming, neutrophil trajectories from systemic preconditioning to intratumoral states, and the evidence linking FOSL2/Fra‐2 to these programs.

### 7.1 TAM‐Stroma Circuits: From Microenvironmental Conditioning to Tissue Remodeling

TAMs are highly plastic components of the tumor ecosystem. Their phenotype reflects not only developmental origin, but also continuous instruction from stromal, vascular, and metabolic niches, allowing macrophage states to diverge across space and over time (Figure 6).

**FIGURE 6:**
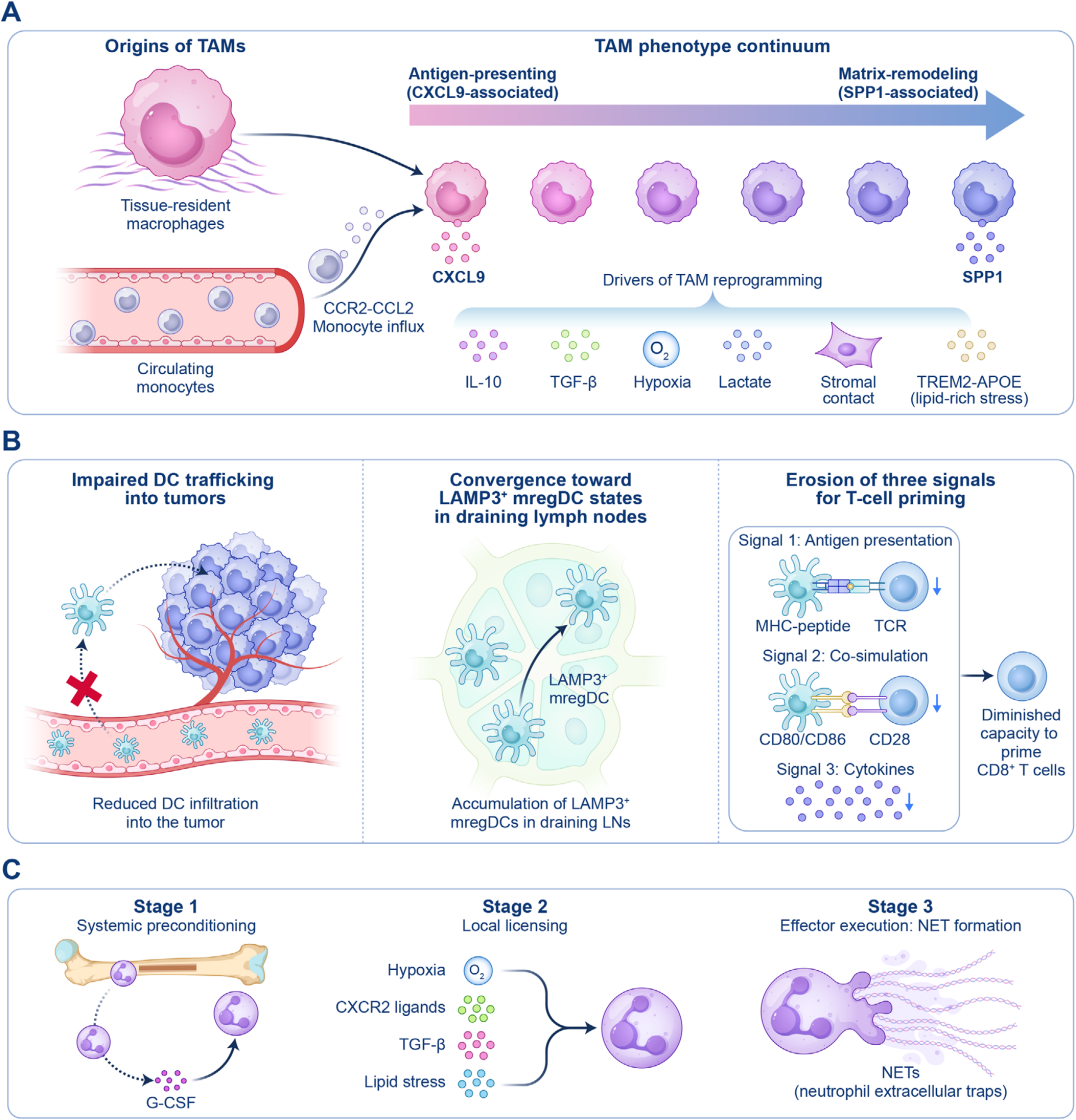
Myeloid and stromal crosstalk in the tumor microenvironment. (A) Tumor‐associated macrophages (TAMs) derive from tissue‐resident macrophages and recruited monocytes and form a niche‐imprinted continuum rather than a fixed M1/M2 state. CCR2–CCL2 supports monocyte influx, whereas interleukin‐10 (IL‐10), transforming growth factor‐β (TGF‐β), hypoxia, lactate, stromal contact, and lipid‐rich TREM2–APOE‐associated stress shape TAM programs along an axis from CXCL9‐associated antigen presentation to SPP1‐associated matrix remodeling. (B) Dendritic cell (DC) function is constrained by trafficking and priming bottlenecks. Reduced DC entry into tumors, convergence toward LAMP3^+^ mature regulatory DC states in draining lymph nodes, and erosion of antigen presentation, costimulation, and cytokine signals collectively impair CD8^+^ T‐cell priming. (C) Neutrophil states are shaped through systemic preconditioning, local licensing, and effector execution. Granulocyte colony‐stimulating factor (G‐CSF)‐driven emergency granulopoiesis expands neutrophils with suppressive or neutrophil extracellular trap (NET)‐prone potential, whereas hypoxia, CXCR2 ligands, TGF‐β and lipid stress license intratumoral neutrophil programs that culminate in NET formation and immune exclusion. DC, dendritic cell; G‐CSF, granulocyte colony‐stimulating factor; IL‐10, interleukin‐10; NET, neutrophil extracellular trap; TAM, tumor‐associated macrophage; TGF‐β, transforming growth factor‐β.

#### 7.1.1 TAM State Diversity and Niche Regulation

TAMs arise from both tissue‐resident macrophages and recruited monocytes, but ontogeny provides only the starting point for their intratumoral identity. CCR2–CCL2 signaling promotes monocyte influx. and CSF‐1–CSF‐1R signaling sustains macrophage survival and differentiation after tumor entry (Figure 6A) [62, 63]. Once within the tumor, these cells are continuously reprogrammed by their surroundings. Single‐cell and spatial profiling have moved the field beyond fixed M1/M2 endpoints, revealing overlapping macrophage states imprinted by cytokines, oxygen tension, nutrient availability, and interactions with malignant and stromal cells [62, 273, 274, 275]. Across tumor types, the CXCL9‐high macrophages tend to express IFN‐responsive and antigen‐presenting programs. SPP1‐high states, by contrast, are enriched for tissue‐remodeling features [276, 277, 278]. These poles capture functional tendencies, not fixed macrophage lineages.

Local signals can redirect recruited monocytes even when they enter tumors with inflammatory features [279, 280]. IL‐10–STAT3 and TGF‐β–SMAD signaling can favor suppressive or tissue‐remodeling functions in selected settings, and reciprocal interactions with fibroblasts and malignant cells can stabilize these states [63, 274, 281]. Metabolism adds another layer of instruction [282, 283]. Hypoxic regions induce HIF‐dependent programs linked to angiogenesis and impaired local T‐cell activity [282, 283, 284]. Tumor‐derived lactic acid can directly alter macrophage function; in experimental tumor models, it induces HIF‐1α‐dependent VEGF and ARG1 expression [283, 285, 286]. Lipid‐rich and highly phagocytic niches are associated with TREM2‐high, often APOE‐expressing macrophage states that display immunosuppressive features in several tumor contexts [279, 287]. These associations should not be mistaken for direct evidence of metabolic flux: metabolite abundance and pathway‐expression signatures identify metabolic states, but substrate use requires functional or isotope‐tracing approaches [283, 285, 286]. TAM identity therefore emerges from the intersection of ontogeny, transcriptional state, metabolism, function, and spatial position. Macrophage‐directed therapies may have limited durability if the niche that sustains suppressive states remains intact [278, 282, 285, 288].

#### 7.1.2 TAM–Stromal Circuits in Matrix and Vascular Remodeling

TAMs help reshape the physical landscape of tumors by acting on both the ECM and the vasculature [63, 289, 290]. Together with fibroblasts, they promote matrix deposition and remodeling; in some tumor models, macrophages themselves also contribute to collagen production. Macrophage‐rich stromal regions can consequently become less permissive to CD8^+^ T‐cell infiltration and function, although the extent of this effect depends on macrophage state and spatial context [63, 291, 292]. The same circuitry extends to the vasculature. TAM‐derived proangiogenic and vasoactive signals can drive disorganized vessel growth, endothelial dysfunction, and poor perfusion, deepening local hypoxia [62, 63, 64, 277, 293]. Fibroblasts and endothelial cells, in turn, reinforce these macrophage programs. The result is a coupled stromal‐vascular niche in which matrix remodeling and vascular dysfunction sustain one another, restricting immune access and preserving hypoxic conditions [62, 275, 292, 294, 295].

### 7.2 Dendritic Cells

#### 7.2.1 DC Trafficking Constraints and mregDC Convergence

After acquiring tumor antigens, cDC1s migrate through CCR7‐dependent routes to tdLNs, where they cross‐prime tumor‐specific T cells (Figure 6B) [296, 297]. This route can fail before priming begins. Effective migration depends not only on CCR7 signaling but also on the cytoskeletal and metabolic machinery required for sustained movement. Tumor‐associated stress can disrupt DC motility, antigen transport, and antigen‐presenting capacity, weakening the link between tumor‐antigen capture and T‐cell priming [298, 299, 300].

DC maturation presents a different challenge [47, 301]. Single‐cell studies have identified a recurrent LAMP3‐associated state termed mature DCs enriched in immunoregulatory molecules (mregDCs). These cells can arise from both cDC1s and cDC2s after antigen uptake and retain migratory features, including CCR7 expression, while acquiring inhibitory molecules such as PD‐L1 and PD‐L2 [47, 301]. This combination resists a simple stimulatory‐versus‐suppressive classification. Their functional output depends on the local balance of activating and inhibitory signals, and the stability and reversibility of this state remain unresolved [301, 302, 303]. A more precise therapeutic goal is to preserve antigen transport and productive T‐cell priming while uncoupling the immunoregulatory programs that accompany mregDC maturation.

#### 7.2.2 Metabolic and Mechanical Regulation of DC Functional States

DC dysfunction in tumors is not simply a consequence of nutrient scarcity. Receptor signaling, lipid handling, and biophysical cues can actively reprogram DC metabolism and influence whether maturation remains immunogenic or becomes regulatory [300, 302]. Mer receptor tyrosine kinase (MerTK) illustrates this principle. Following uptake of apoptotic tumor cells, MerTK signaling increases fatty‐acid oxidation and mitochondrial respiration while dampening DC maturation and T‐cell stimulation [302]. Lipid handling is equally consequential. Excessive lipid accumulation can impair antigen processing and presentation, yet effective cholesterol mobilization is required for DC maturation and productive T‐cell priming [299, 300]. Tumor‐derived metabolites can act through related receptor‐ and metabolism‐dependent pathways to alter antigen presentation and DC state [50]. Mechanical cues provide a distinct route into the same functional decision. The mechanosensitive ion channel Piezo1 senses matrix stiffness and inflammatory signals through calcium‐calcineurin‐NFAT and SIRT1‐HIF‐1α pathways. In experimental models, Piezo1 activity favors IL‐12 production and Th1 differentiation. Its loss shifts the response toward TGF‐β1 production and Treg differentiation [304]. Together, these findings suggest that metabolic and mechanical signals converge on a shared decision point that biases DCs toward immunogenic or regulatory maturation [302, 304].

#### 7.2.3 Three‐Signal Imbalance in DC‐Mediated T‐Cell Priming

Antigen capture and DC maturation do not necessarily translate into productive T‐cell priming. Productive priming requires coordinated antigen recognition, costimulation, and inflammatory cytokine signals [48]. mregDCs illustrate this distinction particularly well [305]. They can retain mature and migratory features while simultaneously expressing inhibitory ligands such as PD‐L1 and PD‐L2 and producing less IL‐12 [301, 303, 306]. Thus, antigen transport and DC maturation may remain intact even as the signals required for productive T‐cell priming become imbalanced. The surrounding myeloid niche can deepen this imbalance. TAM‐derived IL‐10 suppresses IL‐12 production by intratumoral DCs, weakening an important signal for durable CD8^+^ T‐cell responses [48, 49]. Maturation, in other words, is not synonymous with immunogenicity. Restoring productive priming may require preserving DC trafficking and antigen presentation while reducing the inhibitory signals that limit their ability to activate tumor‐specific T cells [48, 302].

### 7.3 Neutrophils

#### 7.3.1 Systemic Preconditioning: Circadian Aging and Emergency Granulopoiesis

Under homeostatic conditions, circulating neutrophils undergo circadian aging and acquire a CXCR4^hi^CD62L^lo^ phenotype that favors bone‐marrow homing and clearance [307]. Cancer disrupts this homeostatic cycle before neutrophils ever reach the tumor. Altered granulopoiesis and mobilization can shift the maturation and activation states of circulating neutrophils, including the emergence of populations with greater NET‐forming or immunosuppressive potential [65]. Tumor‐derived granulocyte colony‐stimulating factor (G‐CSF) is one driver of this systemic rewiring, inducing emergency granulopoiesis and changing the developmental composition of neutrophils released into the circulation [307, 308]. The tumor therefore recruits from an already altered neutrophil pool (Figure 6C). Local signals then impose a second layer of reprogramming, refining these pre‐existing states within the TME [65, 308].

#### 7.3.2 Niche‐Dependent TAN States and Immune Crosstalk

Single‐cell studies have moved the field beyond a strict N1/N2 classification, revealing multiple, partially overlapping tumor‐associated neutrophil (TAN) states across cancers. These states include inflammatory, antigen‐presenting, angiogenic, and immunoregulatory programs [309, 310]. Local cues help determine which of these programs dominate. Hypoxia, CXCR2‐dependent recruitment, TGF‐β signaling, and altered lipid availability can bias neutrophils toward suppressive or tissue‐remodeling states, although these effects vary across tumors and TAN populations [65, 311, 312].

Lipid metabolism provides one of the clearest mechanistic examples of neutrophil‐mediated immune suppression. FATP2 promotes arachidonic‐acid uptake and prostaglandin E2 (PGE2) synthesis in polymorphonuclear myeloid‐derived suppressor cells (PMN‐MDSCs). Genetic or pharmacologic inhibition of FATP2 reduces their suppressive activity and enhances antitumor immunity in preclinical models [65, 307]. This mechanism is best established in PMN‐MDSCs and should not be assumed to apply uniformly to all TAN states. Suppressive neutrophil populations can also inhibit T‐cell activation through ROS, arginase activity, and inhibitory‐receptor–ligand interactions [312, 313].

Other TAN states behave differently. Antigen‐presenting neutrophils can support T‐cell activation, whereas angiogenic and tissue‐remodeling states can alter stromal and vascular compartments. This functional diversity argues against indiscriminate neutrophil depletion. More selective strategies may be needed to remove tumor‐promoting programs while preserving neutrophil states with immune‐supportive functions.

#### 7.3.3 NETs as Spatial Regulators of Immunity

Neutrophil extracellular traps (NETs) can form chromatin‐rich barriers around tumor cells, limiting direct contact with CD8^+^ T cells and NK cells and shielding malignant cells from cytotoxic attack [66, 67]. The result is a form of immune exclusion created by physical separation. NETs can also remodel the ECM. In models of inflammation‐associated metastatic recurrence, neutrophil elastase and MMP9 sequentially cleave laminin, generating matrix cues that activate integrin α3β1 signaling and awaken dormant tumor cells [65, 314]. NETs therefore act through two complementary mechanisms: immune shielding and matrix remodeling. Both may represent therapeutic vulnerabilities. Preclinical studies show that blocking NET formation or promoting NET degradation, including with DNase‐based approaches, can reduce NET‐mediated tumor protection [66, 67, 307].

### 7.4 Case Node: FOSL2/AP‐1 as a Context‐Dependent Regulator of Myeloid and Stromal States

Mechanistic studies implicate FOSL2/Fra‐2 in selected macrophage and fibroblast programs, but its role in other myeloid compartments is less well defined. Direct evidence is strongest in defined macrophage and fibroblast models. Single‐cell associations across broader myeloid populations remain partly inferential and evidence in tumor‐associated DCs is limited [20, 29, 315].

Macrophages provide the clearest myeloid examples. In lung cancer models, β‐catenin induces FOSL2 and suppresses ARID5A, promoting a macrophage program characterized by CD163, MRC1, IL1R1, and TGFB1 expression [316, 317]. FOSL2 knockdown reverses part of this program and reduces the tumor‐supportive activity of these macrophages [316, 317]. A different mechanism operates in glioma. Tumor‐associated monocytes express the EGFR ligands EREG and AREG under FOSL2 control, activating EGFR signaling and mesenchymal programs in neighboring tumor cells [7, 11, 78, 318]. Single‐cell and regulatory‐network analyses have also associated FOSL2 activity with distinct myeloid states, but these associations do not establish that FOSL2 drives their spatial distribution or persistence [316, 317, 319]. How broadly these mechanisms extend across tumor types remains unresolved.

The evidence for tumor‐associated DCs remain limited. FOSL2 has been implicated in DC differentiation and inflammatory responses outside cancer, including regulation of CD11c expression [320, 321, 322, 323]. Direct evidence that FOSL2 controls antigen processing, migration, or T‐cell priming in tumor‐associated DCs is still lacking. Establishing such a role will require lineage‐specific perturbation coupled to measurements of antigen presentation, trafficking, and T‐cell activation [322, 324, 325].

CAFs provide a second compartment with direct mechanistic evidence. ANKRD1 associates with c‐JUN and FOSL2 and facilitates AP‐1 complex formation at myofibroblastic CAF regulatory regions. Loss of ANKRD1 reduces expression of CAF effector genes including ACTA2, COL1A1, and HAS2 and weakens the c‐JUN/FOSL2 interaction [27, 315, 326]. Breast CAFs reveal a distinct FOSL2 program. Estrogen–cAMP–PKA signaling increases FOSL2, which directly activates Wnt5a. CAF‐derived Wnt5a then engages endothelial FZD5–NF‐κB–ERK signaling and promotes VEGF‐independent angiogenesis [29]. These studies establish direct roles for FOSL2 in defined stromal programs. Whether the same mechanisms drive immune exclusion or treatment resistance remains to be tested.

## 8 Therapeutic Implications: Matching Intervention to Immune‐Dynamic Failure Modes

TME‐directed therapy should address the dominant barrier to antitumor immunity, not target expression alone. Tumors with effector cells trapped at stromal margins require a different strategy from those in which immune cells enter but fail to recognize tumor antigens or sustain effector function. Progression and treatment can add another layer of resistance by generating fibrotic, hypoxic, or myeloid‐rich niches. These tissue states may persist even when the original immune defect is corrected.

We organize these therapeutic challenges around four major failure modes: failed immune access, defective antigen visibility and priming, failure to sustain effector function, and suppressive tissue remodeling. These modes can coexist and evolve over time. Each points to a different set of biomarkers and therapeutic priorities.

### 8.1 Restoring Tumor‐Nest Access

Access‐limited tumors are defined by a spatial mismatch: effector cells are present but fail to reach malignant nests or maintain productive contact with tumor cells. Total immune‐cell abundance can obscure this defect. Restoring access first requires identifying where movement breaks down, at recruitment, vascular extravasation, or transit through stromal tissue. Depending on the dominant barrier, therapeutic strategies may include vascular normalization, modulation of CAF or ECM barriers, and targeting of CXCL12–CXCR4 or TGF‐β‐associated stromal programs. Success should be judged by effective tumor‐cell engagement, not simply by an increase in infiltrating immune cells. FOSL2‐dependent CAF programs may contribute to stromal and vascular barriers [29, 326]. Direct evidence that FOSL2 drives T‐cell exclusion, or that its inhibition restores immune access, is still lacking.

### 8.2 Restoring Priming and Antigen Visibility

Even when immune cells reach tumor nests, antitumor immunity can fail if malignant cells remain poorly visible or if DC priming is ineffective. This barrier involves two linked defects: reduced tumor‐cell visibility and impaired APC competence. Tumor‐cell visibility depends on MHC‐I/APM integrity, IFN pathway competence, and, for iNKT‐based approaches, CD1d‐dependent lipid‐antigen presentation. APC competence depends on DC activation, migration, costimulation, and inflammatory cytokine support for effective priming. Evidence for this barrier may include MHC‐I/APM loss, defective IFN‐response signatures, reduced or mislocalized cDC1s, inhibitory‐dominant mregDC states, weak IL‐12‐associated signaling, and the absence of mature or well‐organized TLS and APC–T‐cell interaction niches [42, 228, 251, 252]. This axis remains double‐edged because IFN‐driven antigen visibility can be coupled to PD‐L1 induction, while DC maturation can coexist with immunoregulatory signaling, and broad immune activation may increase inflammation without generating durable priming. AP‐1/FOSL2‐associated rewiring has been linked to tumor‐cell IFN‐response and immune‐regulatory programs in selected preclinical models [32, 327, 328, 329, 330]; however, direct evidence that FOSL2 controls MHC‐I antigen presentation in vivo remains limited. Therefore, priming‐directed combinations should be prioritized when low antigen visibility or defective APC function is documented, rather than being used as generic immune stimulants.

### 8.3 Sustaining Effector Function Under Inhibitory and Metabolic Stress

The third mode of therapeutic failure occurs when immune cells gain tumor access but cannot maintain effective function within the TME. Inhibitory signaling, metabolic and mitochondrial stress, epigenetic stabilization of exhaustion, and lineage reprogramming can all contribute to this failure [260, 331]. Potential interventions include checkpoint blockade, metabolic targeting, cytokine or costimulatory support, and engineered lymphocyte platforms designed to preserve effector function [260]. Candidate biomarkers include hypoxia, mitochondrial fitness, inhibitory‐receptor expression combined with functional‐state markers, exhaustion‐associated chromatin features, and lineage reprogramming [260, 331]. These strategies may have limited benefit when immune access or the required recognition pathway is absent. When metabolic dysfunction predominates, treatment should target the specific metabolic constraint rather than rely on checkpoint blockade alone [264, 332, 333].

### 8.4 Limiting Suppressive Tissue Remodeling

Tumor progression and treatment can create fibrotic, hypoxic, senescent, NET‐rich, and myeloid‐dominant niches that suppress antitumor immunity [334, 335]. These changes can promote immune exclusion, metabolic stress, and treatment resistance [59, 105]. Therapeutic strategies may therefore need to target CAF–TAM interactions, persistent SASP signaling, NET accumulation, vascular dysfunction, and hypoxia‐related stress, rather than depleting individual suppressive cell populations alone. Candidate biomarkers include ECM deposition, suppressive TAM or TAN states, NETs, SASP factors, hypoxia signatures, and treatment‐associated expansion of stromal or myeloid compartments. FOSL2/Fra‐2 has been implicated in selected fibroblast and macrophage programs, but its contribution remains context dependent and requires direct functional validation.

## 9 Evidence Map and Knowledge Gaps

Increasingly detailed immune‐state maps have outpaced mechanistic understanding of how these states arise and function within tumors. Progress now requires linking cellular states to spatial context, upstream regulators, and measurable immune functions. These relationships should be resolved by compartment, niche location, model system, and functional endpoint. We therefore organize FOSL2/Fra‐2‐associated programs by compartment and level of evidence, rather than as a unified pan‐TME pathway (Table 2). This framework distinguishes perturbation‐supported mechanisms from context‐dependent associations. Stronger evidence for causality comes from combining state mapping with experimental perturbation and functional readouts, including trafficking, antigen presentation, cytokine production, cytotoxicity, and matrix remodeling.

**TABLE 2:** Evidence map for FOSL2/Fra‐2‐associated programs in the TME.

Compartment	FOSL2/Fra‐2‐associated program	Evidence	Interpretation
Malignant cells	KRAS‐, hypoxia‐, and stress‐linked programs; chemokine output; ANXA1‐mediated macrophage recruitment; ferroptosis resistance.	Mechanistic and human correlative evidence [30, 31, 78, 125, 126, 315].	Context‐dependent; often immune‐restrictive.
CD8^+^ T cells	AP‐1 remodeling during chronic stimulation and exhaustion‐associated transitions.	Strong AP‐1 evidence; limited FOSL2‐specific validation [51, 54, 57, 172, 176, 197, 198].	Causal role of FOSL2 remains uncertain
Tregs and CD4^+^ T‐cell states	Treg development, helper‐cell differentiation, and tumor‐adapted AP‐1 programs.	Direct FOSL2/Fra‐2 and broader AP‐1 evidence [214, 216, 219, 222].	Bidirectional and context‐dependent
B cells and TLSs	B‐cell development and FOS/AP‐1‐associated intratumoral B‐cell states.	Developmental and associative evidence [25, 245].	Direct TLS regulation remains unproven.
NK and iNKT cells	Lineage development and maturation.	Direct genetic/developmental evidence [24, 271, 272].	Tumor‐specific roles remain unclear.
Myeloid cells and TAMs	Macrophage recruitment, polarization, and suppressive remodeling.	Mechanistic and single‐cell evidence [78, 316, 317].	Relevant to myeloid‐rich and hypoxic TMEs.
Dendritic cells	AP‐1/Fra‐2‐associated transcriptional regulation.	Mostly nontumor evidence [322, 325].	Tumor DC functions remain unresolved.
CAFs and stromal niches	Fibroblast activation, matrix remodeling, and angiogenic programs.	Fibrosis and tumor‐CAF evidence [27, 29, 321, 326].	Potential role in stromal exclusion.

*Note*: Evidence strength and directionality are model‐dependent. FOSL2/Fra‐2 is presented as a context‐dependent AP‐1 node rather than a universal driver or therapeutic target.

Abbreviations: AP‐1, activator protein 1; CAF, cancer‐associated fibroblast; DC, dendritic cell; iNKT, invariant natural killer T; NK, natural killer; TAM, tumor‐associated macrophage; TLS, tertiary lymphoid structure; TME, tumor microenvironment; Treg, regulatory T cell.

Despite this progress, several gaps continue to limit translation. Much of the strongest mechanistic evidence for immune‐state regulation still comes from murine genetic models and reductionist in vitro systems. For FOSL2, evidence from human tumors remains limited, especially when cell state and spatial organization are considered together. Defining its role will require integrated single‐cell and spatial analyses across tumor compartments. More broadly, transcriptional regulators cannot be interpreted independently of cellular context. Their effects vary with lineage, activation state, cytokine exposure, and tissue environment, even among closely related T‐cell populations.

A third limitation concerns regulatory specificity. For FOSL2/AP‐1, it remains unclear how AP‐1 partner composition, including alternative JUN‐family partners, interacts with lineage‐defining transcription factors, metabolic signals, and chromatin accessibility to determine genomic binding and transcriptional output in different cell states. Resolving these questions will require integration of single‐cell multiomics with direct mapping of transcription‐factor occupancy and enhancer activity, together with functional perturbation to distinguish causal regulation from state association.

## 10 Conclusion and Prospects

The success of antitumor immunity depends not only on immune‐cell infiltration but also on spatial positioning, tumor recognition, maintenance of effector function, and the surrounding tissue environment. These processes support a failure‐mode‐based approach to TME‐directed therapy rather than nonspecific immune activation. Combination strategies should therefore be guided by the dominant barrier in each tumor, together with biomarkers of spatial organization, antigen presentation, effector competence, and tissue remodeling.

FOSL2/Fra‐2 illustrates a broader principle in TME biology: transcriptional regulators rarely function as uniformly pro‐ or antitumor drivers. FOSL2‐associated programs have been linked to stromal activation, myeloid remodeling, stress responses, and selected immune‐regulatory processes, but these observations should not be collapsed into a single pan‐TME pathway. Its value in this review is therefore not as a universal therapeutic target, but as an example of why transcription‐factor activity must be interpreted according to cell type, tissue context, and experimental evidence.

Future TME research should connect increasingly detailed spatial and single‐cell maps to causal mechanisms and therapeutic response. Progress will require longitudinal sampling, compartment‐specific perturbations, chromatin and transcription‐factor mapping, and functional immune endpoints within clinically annotated cohorts. The central task is to distinguish causal from reactive cell states and determine which states can be safely and durably reprogrammed in patients.

## 11 Literature Search Strategy

We performed a structured literature search in PubMed/MEDLINE, Web of Science, and Google Scholar, covering literature published up to February 1, 2026. Searches used Medical Subject Headings (MeSH) where applicable and free‐text keywords spanning core concepts (TME; immune‐cell dynamics; cancer therapy) and major cellular and mechanistic modules (T‐cell exhaustion; regulatory T cells; TAMs; CAFs; DCs; NK cells; iNKT cells; hypoxia and metabolism; SASP and senescence; antigen presentation including MHC‐I; IFN signaling), together with the focal transcriptional regulator (AP‐1; FOSL2/Fra‐2).

We prioritized primary research and high‐quality review articles, emphasizing recent work (particularly within the preceding 5 years) while retaining older landmark studies that established key concepts and methods. Studies were included if they provided mechanistic evidence or clinically relevant associations related to immune and stromal dynamics in the TME, supported by defined model systems and interpretable readouts (e.g., spatial localization, single‐cell profiling, functional perturbation, or treatment response).

We excluded studies with insufficient methodological details, unvalidated non–peer‐reviewed reports, and findings not directly relevant to immune‐cell dynamics in tumors. Evidence was evaluated according to both mechanistic strength and clinical relevance rather than by a single fixed hierarchy. Direct genetic or pharmacologic perturbation with functional validation was considered the strongest evidence for causality, whereas well‐controlled human studies, including spatial and single‐cell analyses, were given greater weight for clinical and biological relevance. Descriptive or in vitro findings were considered hypothesis‐generating unless supported by independent functional validation or replication across models or cohorts.

## Author Contributions

Pin Guo, Yizhao Xie, and Rui Song contributed equally to visualization, data analysis, and manuscript drafting. Mengdi Yang, Zhen Lin, Chao Guan, Zechen Wang, and Jin Wen contributed to visualization, data collection, and technical support. Jin Wen coordinated the research process. Guang‐Liang Chen contributed to visualization and data analysis, supervised the study, and wrote and revised the manuscript. All the authors have reviewed and approved the final manuscript.

## Funding

This study was supported by the National Natural Science Foundation of China (Nos. 82573874, 81802362, and 82303398), Shanghai Sailing Program (22YF1408700), and FUSCC‐Physician Excellence Program (ZYJH202109).

## Ethics Statement

The authors have nothing to report.

## Conflicts of Interest

The authors declare no conflicts of interest.

## Data Availability

Data sharing is not applicable to this article because no datasets were generated or analyzed in the current study.
